# RIP1 inhibition blocks inflammatory diseases but not tumor growth or metastases

**DOI:** 10.1038/s41418-019-0347-0

**Published:** 2019-05-17

**Authors:** Snahel Patel, Joshua D. Webster, Eugene Varfolomeev, Youngsu C. Kwon, Jason H. Cheng, Juan Zhang, Debra L. Dugger, Kate E. Wickliffe, Allie Maltzman, Swathi Sujatha-Bhaskar, Pawan Bir Kohli, Sreema Ramaswamy, Gauri Deshmukh, Bianca M. Liederer, Rina Fong, Greg Hamilton, Patrick Lupardus, Patrick Caplazi, Wyne P. Lee, Menno van Lookeren Campagne, Adam Johnson, Brent S. McKenzie, Melissa R. Junttila, Kim Newton, Domagoj Vucic

**Affiliations:** 1 Department of Discovery Chemistry, Genentech, 1 DNA Way, South San Francisco, CA 94080 USA; 2 Department of Pathology, Genentech, 1 DNA Way, South San Francisco, CA 94080 USA; 3 Department of Early Discovery Biochemistry, Genentech, 1 DNA Way, South San Francisco, CA 94080 USA; 4 Department of Translational Immunology, Genentech, 1 DNA Way, South San Francisco, CA 94080 USA; 5 Department of Molecular Oncology, Genentech, 1 DNA Way, South San Francisco, CA 94080 USA; 6 Department of Physiological Chemistry, Genentech, 1 DNA Way, South San Francisco, CA 94080 USA; 7 Departments of Immunology, Genentech, 1 DNA Way, South San Francisco, CA 94080 USA; 8 Department of Biochemical and Cellular Pharmacology, Genentech, 1 DNA Way, South San Francisco, CA 94080 USA; 9 Department of Drug Metabolism and Pharmacokinetics, Genentech, 1 DNA Way, South San Francisco, CA 94080 USA; 10 Department of Structural Biology, Genentech, 1 DNA Way, South San Francisco, CA 94080 USA; 11 Present Address: Gilead, Foster City, CA 94404 USA; 12 Present Address: Amgen, South San Francisco, CA 94080 USA

**Keywords:** Cell death and immune response, Acute inflammation

## Abstract

The kinase RIP1 acts in multiple signaling pathways to regulate inflammatory responses and it can trigger both apoptosis and necroptosis. Its kinase activity has been implicated in a range of inflammatory, neurodegenerative, and oncogenic diseases. Here, we explore the effect of inhibiting RIP1 genetically, using knock-in mice that express catalytically inactive RIP1 D138N, or pharmacologically, using the murine-potent inhibitor GNE684. Inhibition of RIP1 reduced collagen antibody-induced arthritis, and prevented skin inflammation caused by mutation of *Sharpin*, or colitis caused by deletion of *Nemo* from intestinal epithelial cells. Conversely, inhibition of RIP1 had no effect on tumor growth or survival in pancreatic tumor models driven by mutant Kras, nor did it reduce lung metastases in a B16 melanoma model. Collectively, our data emphasize a role for the kinase activity of RIP1 in certain inflammatory disease models, but question its relevance to tumor progression and metastases.

## Introduction

Aberrant cell death contributes to immune disorders, tissue damage, cancer, and neurodegeneration [1–3]. Apoptosis is a regulated form of cell death orchestrated by a family of cysteine proteases called caspases [4], whereas necroptosis is a caspase-independent death program mediated by the kinase RIP3 and the pseudokinase MLKL (mixed-lineage kinase domain-like) [3, 5]. The kinase RIP1, acting downstream of TNFR1 (tumor necrosis factor receptor 1), can trigger apoptosis through binding to FADD, the activating adaptor for caspase-8, or necroptosis through binding to RIP3. It also contributes to the activation of NF-κB and MAPK signaling by TNFR1 [5–11].

Although the kinase activity of RIP1 is dispensable for NF-κB and MAPK signaling by TNFR1, it is essential for interactions between RIP1 and RIP3 that activate RIP3 to phosphorylate MLKL. Oligomerization and translocation of MLKL to cell membranes results in cell lysis [5, 12, 13]. In some situations, such as genetic ablation of NEMO (NF-κB essential modulator) [14–16], activation of RIP1 instead triggers apoptosis [17, 18]. Genetic studies in mice implicate this cell death in inflammation. For example, the *Cpdm* mutation, which inactivates the Sharpin subunit of LUBAC (linear ubiquitin chain assembly complex) [6], causes multi-organ inflammation that is dependent on both TNF and the kinase activity of RIP1 [19–21]. Other studies have implicated the kinase activity of RIP1 in ischemia-reperfusion injury and neurodegeneration/neuroinflammation [2, 22–24].

Recently, the kinase activity of RIP1 was shown to limit anti-tumor immunity in pancreatic cancer models [25, 26]. Inhibition of RIP1 suppressed tumor growth by eliciting a highly immunogenic myeloid and T-cell infiltrate [25], due to the reprogramming of tumor-associated macrophages (TAMs) to an M1-like phenotype [26]. Independent studies have claimed that inhibition of RIP1 prevents tumor cell metastasis [27, 28].

Given the potential therapeutic benefit of inhibiting RIP1, selective RIP1 inhibitors have been reported [29–32], but most cannot be used in mouse models because they target human RIP1 more effectively than mouse RIP1 and/or they have suboptimal pharmacokinetic properties [29–31, 33]. We have developed GNE684 as a potent inhibitor of murine RIP1 that is suitable for multi-day dosing. It provided comparable protection to genetic inactivation of RIP1 against colitis triggered by *Nemo* deficiency, collagen antibody-induced arthritis, and *Cpdm*-associated skin inflammation. Importantly, inhibition of RIP1, either genetically or chemically, had no effect on the growth of pancreatic tumors or on melanoma metastasis. Therefore, targeting the kinase activity of RIP1 appears to have more potential as an intervention strategy in inflammatory diseases than in cancer.

## Methods

### Reagents and antibodies

Human recombinant TNF, Nec-1a, BV6, GNE684, and GSK547 were all synthesized at Genentech. The primary antibodies used were directed against: RIP1 (610459), pJNK (562480) (BD Biosciences); IκBα (9242), HSP90 (4874), A20 (5630), caspase-8 (9746), human pRIP1 S166 (65746), and RIP3 (13526) (Cell Signaling Technology); mouse pMLKL S345 (ab196436) and human c-IAP2 (ab32059) (Abcam); MLKL (MABC604) (Millipore); mouse c-IAP2 and RIP3 (Genentech).

### Synthesis of GNE684

The complete synthesis of GNE684 is reported in the supplement.

### Cell lines

Human colon carcinoma HT-29, T-cell Jurkat, mouse monocyte J774A.1, macrophage RAW 264.7, fibroblast L929 and rat myoblast H9c2 cell lines were from ATCC; EA1-transformed MEFs from Genentech; human esophageal OE19 cell lines from ECACC; human stomach SNU-620 cell line from KCLB. Primary human colon, stomach, cynomolgus monkey colon, stomach, porcine colon, stomach, mouse colon, esophagus and small intestine epithelial cells were purchased from Cell Biologics (IL, USA).

### Viability assays

Cell viability was assessed using Cell TiterGlo (Promega) following the manufacturer’s specifications.

### Western blot analysis and immunoprecipitation

Western blot analyses and immunoprecipitations were performed with the following buffer: 1% Triton X-100, 25 mM Tris-HCl buffer (pH 7.5), 150 mM NaCl, 1 mM EDTA, Halt Protease and Phosphatase Inhibitor Cocktail (Thermo Scientific). Cells were lysed on ice for 30 min and centrifuged at 14,000 rpm for 10 min at 4 °C. Immunoprecipitations were performed over night at 4 °C with anti-Caspase-8 antibody (5F7, Enzo Scientific) and protein A/G beads. Immunoprecipitated protein complexes were washed several times in lysis buffer, resolved on SDS-PAGE and immunoblotted with the indicated antibodies.

### Mice for animal studies

Ripk3^-/-^ [34], Ripk1^KD/KD^ [12], A20^fl/fl^ [22], ATG16L1^fl/fl^ [35], Cpdm [36], Nemo^fl/fl^ [37], *Villin*.cre [38], and *Villin*.CreERT2 [39] mice were described previously. All animals were dosed and monitored according to guidelines from the Institutional Animal Care and Use Committee (IACUC) on study protocols approved by the Laboratory Animal Resource Committee at Genentech. Whenever possible, littermates were used, and all animals were randomized during group allocation. Pathologists assessed the samples in a blinded fashion. All data were analyzed by appropriate statistical tools (listed with the description of different methods/models) and all experiments included control groups. All individuals participating in animal care and use were required to undergo training by the institution’s veterinary staff.

### TNF-induced SIRS

Littermates of both sexes were dosed with murine TNF (300 μg/kg)(R&D Systems) and zVAD-FMK (10 mg/kg)(APExBIO) intravenously (IV) via the tail vein. GNE684 (indicated amounts formulated in 10% DMSO/MCT) was administered PO. Body temperature was determined after 2 and 4 h by measuring the temperature of the skin in the abdominal area using Braun ThermoScan PRO 4000 Infrared Ear Thermometer. Mice with a body temperature below 23.6 °C or that were moribund were euthanized. Statistical analyses were done using Jump (Oneway analysis with Dunnett’s Method).

### NEMO deletion induced colitis and ileitis

*Nemo*^*fl/fl*^ *Villin*.creERT2 mice (NEMO IEC-KO) [17] were treated with tamoxifen (80 mg/kg, IP) on days 1–3 to induce NEMO deletion. When treated with indicated amounts of GNE684 (BID (twice daily), PO (per os), in 10% DMSO/MCT), mice were dosed on days 2–6. Serum, plasma, ileum, and colon were collected for PK and cytokine analyses and histology. Large and small intestinal sections were visually separated into three regions and each region was scored independently according to the following matrixes. Scores of each segment were then summed for final large and small intestinal histology scores. Large intestine: (0) Within normal limits, (1) Few focal inflammatory foci with or without individual pyknotic cells in crypts, (2) Multifocal, discrete crypt degeneration with associated inflammation, (3) Moderate, multifocal crypt loss and inflammation with or without ulceration, (4) Multifocal, locally extensive inflammation with crypt loss and ulceration, (5) Extensive, confluent inflammation with crypt loss and inflammation. Small intestine: (0) No pyknotic cells observed, (1) Rare pyknotic cells in crypts, (2) Mild, multifocal pyknotic cells in crypts with minimal disruption of crypt architecture, (3) Moderate, multifocal pyknotic cells in crypts with disruption of crypt architecture, Paneth cell loss, and variable neutrophilic inflammation, (4) Moderate, multifocal crypt cell pyknosis and extensive suppurative inflammation.

### Collagen antibody-induced arthritis (CAIA) model

Female mice aged 8 weeks received 2 mg of a cocktail of monoclonal anti-collagen antibodies (Chondrex, Inc.) by IV in sterile PBS on day 0, followed by 50 μg LPS IP in PBS on day 3. Mice were monitored for 10 days as previously described [40]. For the treatment experiment mice were dosed on days 4–9 with vehicle (10% DMSO/MCT), anti-ragweed-IgG2a (150 μg), mouse TNFR2-IgG2a (150 μg), or GNE684 (50 mg/kg, BID, PO, in 10% DMSO/MCT).

### Sharpin mutation (*Cpdm*) induced skin inflammation

*Cpdm* mice (Jackson Laboratories) were left untreated or treated with GNE684 (50 mg/kg, BID, PO) for 4.5 days. Dorsal and ventral cervical tissues were collected for histology. Histologic lesions in *Cpdm* mice were scored according to the following criteria for inflammation, epidermal hyperplasia, and ulceration/serocellular crusts. The three individual scores were summed for a final score. Inflammation: (1) Slight, multifocal increase in dermal cellularity, (2) Mild to moderate, multifocal increase in dermal cellularity + /- fibrosis, (3) Diffuse, mild to moderate increase in dermal cellularity and fibrosis, (4) Moderate, diffuse increase in dermal cellularity and fibrosis. Epidermal hyperplasia: (1) Multifocal, 2–3 cell layer epidermal thickening, (2) Approximately 1–3 foci of > 3 cell layer expansion of the epidermis, (3) > 2 foci of locally extensive areas of epidermal expansion beyond 3 layers, (4) Extensive epidermal expansion > 3 layers. Ulceration/ serocellular crusts: (1) 1–2 serocellular crusts and/or increased individual pyknotic cells in the epidermis, (2) Single ulcer < 2 follicles in size or > 2 serocellular crusts, (3) Single ulcer > 2 follicles in size or 2–5 ulcers < 2 follicles in size, (4) Multiple ulcers > 2 follicles in size.

### Genetically engineered mouse models of pancreatic cancer

We obtained mice from the following institutions: *Kras*^*LSL–G12D*^ and *Trp53*^*LSL.R270H*^ are from Tyler Jacks (Massachusetts Institute of Technology), *p16/p19*^*fl/fl*^ from Anton Berns (NKI, The Netherlands) and *Pdx1-Cre* from Andy Lowy (University of Ohio). All animals were maintained on a C57BL/6 background. Equal numbers of male and female animals were used for experimental cohorts, dosing commenced following confirmation of tumor burden via ultrasound imaging and animals were equally distributed to treatment arms based on their baseline tumor volumes. All chosen dosing regimens were well tolerated in the Genetically engineered mouse models (GEMMs). Noninvasive imaging and assessment of overall survival were performed as previously described [41]. Animals were monitored daily while on treatment and weights were measured at least weekly. Date of death was based either on mortality or pre-determined morbidity criteria for euthanasia. If deemed moribund, animals were euthanized within 1–4 h. Treatment of mice was continuous until all animals were terminated. Necrostatin (Nec-1a) and GNE684 were dosed at 50 mg/kg, PO, BID (90% methylcellulose, 10% DMSO) until the end of study. Gemcitabine (Gemzar) was dosed IP at 50 mg/kg every 3 days until end of study, as previously reported [42]. Serial ultrasound measurements were used to calculated the difference in log-scale daily fold change between treatment groups, verified by Dunnett’s test (PMID:25376606).

## Results

### GNE684 is a potent cross-species inhibitor of RIP1

**Fig. 1:**
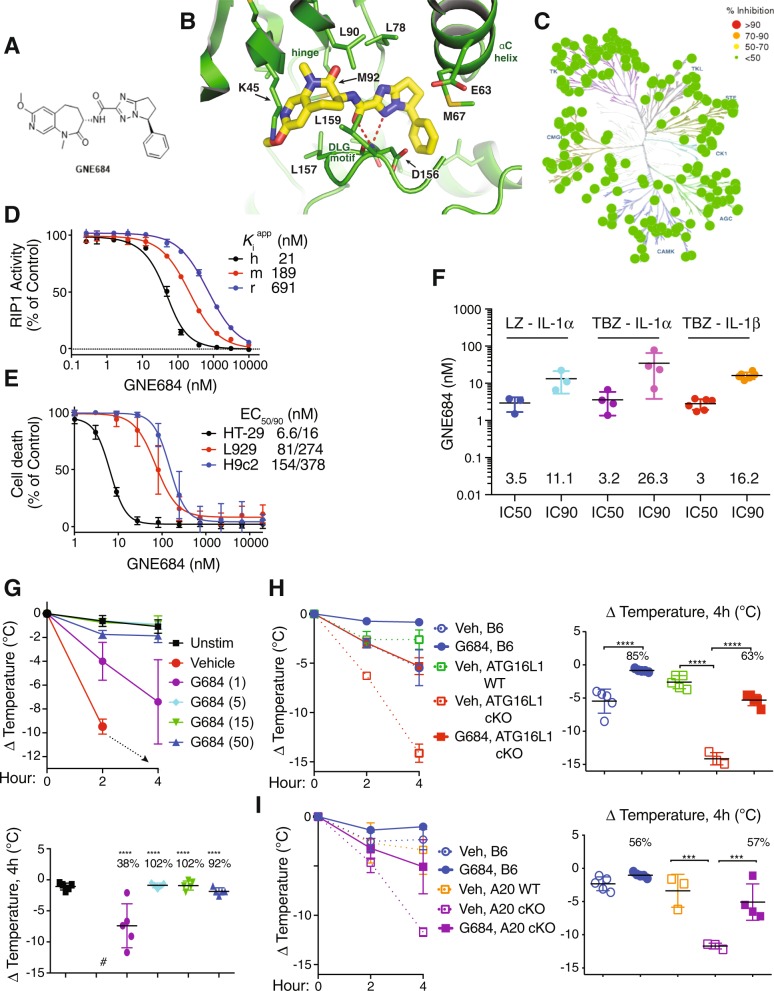
GNE684 is a potent cross-species inhibitor of RIP1. **a** Chemical structure of GNE684. **b** Structure of GNE684 in complex with RIP1 kinase domain. GNE684 is shown as a stick model in yellow, and RIP1 is shown in green. Relevant pocket residues are labeled in addition to the locations of the kinase hinge, αC helix, and DLG motif. **c** Kinase selectivity of GNE684 determined in SelectScreen panel. **d** Inhibition of human (h), mouse (m), and rat (r) RIP1 kinase domain catalytic activity by GNE684 was determined using an assay of RIP1-catalyzed ATP hydrolysis. Data points plotted are the means and standard deviations of four titrations. The mean *K*_i_^app^ values are shown. **e** Inhibition by GNE684 of necroptotic cell death in human HT-29, rat H9c2, and mouse L929 cells treated 18–24 h with TNF (20 ng/ml for HT-29 and H9c2 or 1 ng/ml for L929 cells), BV6 (2 μM for HT-29 and H9c2 cells), and zVAD (20 μM). GNE684 was tested in four (HT-29), eight (H9c2), and six (L929) titrations and the data plotted are the means and standard deviations of all titrations. The mean EC50 and EC90 values are given. **f** Whole-human blood was stimulated with LPS (1 μg/ml) and zVAD (20 μM) (LZ), or TNF (200 ng/ml), BV6 (2 μM), and zVAD (20 μM) (TBZ) for 16 h in the absence or presence of GNE684. Levels of released IL-1α and IL-1β were measured by ELISA. The mean IC50 and IC90 values (nM) are listed above the *x*-axis. **g** Mice were treated with TNF (300 μg/kg) and zVAD (10 mg/kg) in the absence or presence of indicated amounts of GNE684 (G684) (mg/kg). Top graph depicts body temperature measurements at 2 and 4 h after dosing. Bottom graphs depict percent inhibition by indicated doses of GNE684. Animals in the vehicle group were sacrificed before the end of the study (#). **h** Wild-type (B6), and *Villin*.cre (ATG16L1 WT) or *ATG16L1*^*fl/fl*^ *Villin*.cre (ATG16L1 cKO) mice were treated with TNF (300 μg/kg) and, where indicated, with GNE684 (50 mg/kg). **i** Wild-type (B6), and *A20*^*+/+*^ *Villin*.cre (A20 WT) or *A20*^*fl/fl*^ *Villin*.cre (A20 cKO) mice were treated with TNF as in **h** and, where indicated, with GNE684 (50 mg/kg). In **h** and **i** graphs on the left side depict body temperature measurements at 2 and 4 h after dosing, and graphs on the right side depict percent inhibition by GNE684 at 4 h. For **g–i** three asterisks indicate *p* ≤ 0.001 and four *p* ≤ 0.0001 relative to the same strain vehicle controls

To investigate the potential therapeutic benefit of inhibiting RIP1, we developed GNE684 or (S)-N-((S)-7-methoxy-1-methyl-2-oxo-2,3,4,5-tetrahydro-1H-pyrido[3,4-b]azepin-3-yl)-5-phenyl-6,7-dihydro-5H-pyrrolo[1,2-b][1,2,4]triazole-2-carboxamide with cross-species potency against RIP1, exquisite kinase selectivity, and favorable pharmacological properties (Fig. 1a–h, S1, Tables S1 and S2, SI file 1). A co-crystal structure showed that GNE684 binds to the same hydrophobic pocket within the kinase domain of RIP1 that is bound by necrostatins [32] (Fig. 1b, S1, and Table S1). GNE684 binds to an inactive conformation of RIP1, similar to type II kinase inhibitors, with the Asp156 and Leu157 of the DLG motif (commonly DFG in other kinases) in the “out” conformation and the αC helix swung away from the ATP-binding cleft, and lacking the canonical ion pair between the catalytic lysine (Lys45) and αC glutamate (Glu63) (Fig. 1b). GNE684 inhibited human RIP1 potently in vitro, and mouse and rat RIP1 with slightly less potency (Fig. 1d). Accordingly, it inhibited TNF-induced necroptosis in human HT-29, mouse L929, or rat H9c2 cells (Fig. 1e). The cellular potency of GNE684 was confirmed in a human whole blood assay using TBZ (TNF, IAP antagonist BV6, and pan-caspase inhibitor zVAD) or LZ (LPS plus zVAD) to trigger necroptosis and the release of IL-1α and IL-1β (Fig. 1f).

We evaluated the potency of GNE684 in vivo using a model of SIRS (systemic inflammatory response syndrome) that is based on the administration of TNF plus zVAD. Hypothermia in wild-type (WT) mice was almost completely prevented by dosing with 5, 15, or 50 mg/kg of GNE684, while dosing with 1 mg/kg reduced the temperature loss by 38% (Fig. 1g and S1i). We also examined mice lacking *Atg16l1* or *A20* in intestinal epithelial cells (IECs) because these genes encode important negative regulators of TNF-induced cell death, and their mutation is associated with colitis in humans [43, 44]. As expected, ATG16L1 IEC cKO mice and A20 IEC cKO mice exhibited more severe hypothermia after TNF treatment than control mice (Fig. 1h, i). Importantly, hypothermia in both strains was blocked by GNE684 (Fig. 1h, i). Collectively, these data identify GNE684 as an effective inhibitor of RIP1 both in vitro and in vivo.

### GNE684 inhibits RIP1 kinase driven cell death

**Fig. 2:**
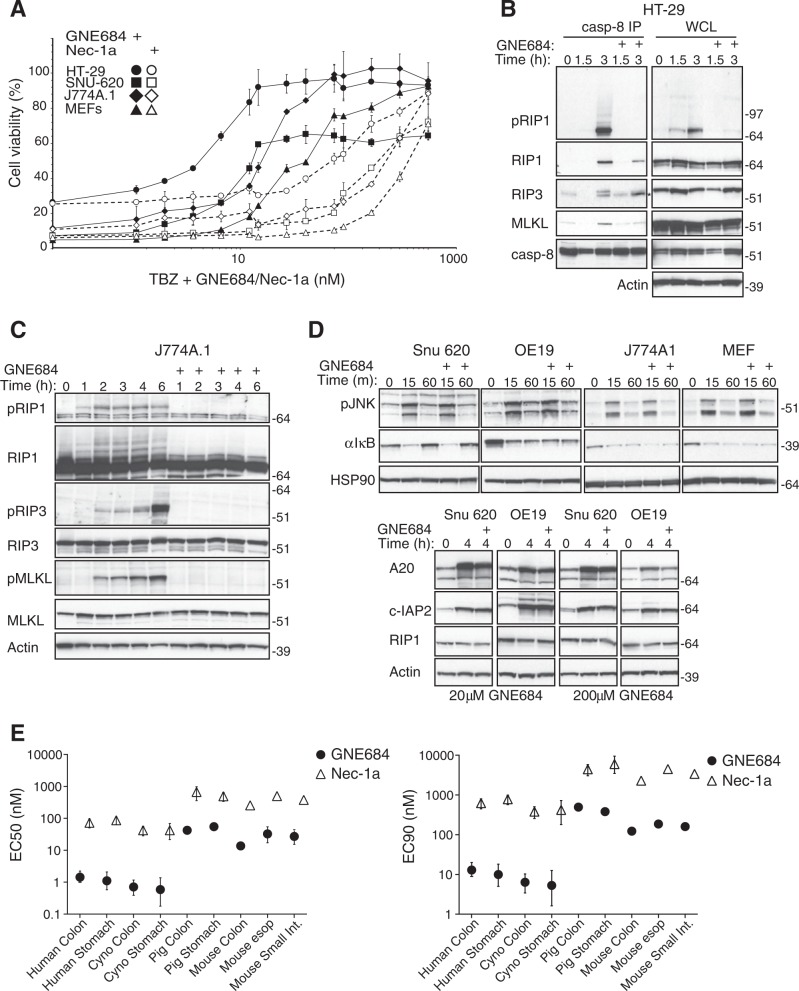
GNE684 inhibits RIP1 kinase driven cell death. **a** Indicated cell lines were treated for 20 h with TNF (20 ng/ml), BV6 (2 μM), and zVAD (20 μM) with increasing amounts of GNE684. Cell viability was assessed by CellTiter-Glo assay. **b**, **c** HT-29 (**b**) and J774A.1 (**c**) cells were treated with BV6 (2 μM), TNF (20 ng/ml), and zVAD (20 μM) with 0 or 20 μM GNE684 for indicated periods of time. Cell lysates were immunoprecipitated with anti-caspase-8 antibodies **b.** Cellular lysates and caspase-8-associated complexes were examined by western blotting with the indicated antibodies. Data are representative of three experiments. **d** Indicated cell lines were treated by TNF (20 ng/ml) with or without GNE684 (20 μM top panels, 20 or 200 μM lower) for indicated periods of time. Cellular lysates were examined by western blotting with the indicated antibodies. Data are representative of three experiments. **e** Indicated primary cells were treated with BV6 (4 μM) and TNF (20 ng/ml) (mouse esophageal and small intestine cells), plus zVAD (20 μM) (all other cells) with increasing amounts of GNE684 or Nec-1a. GNE684 and Nec-1a EC50s and EC90s were calculated using Prism 8 software using data of at least two independent experiments. Error bars indicate standard deviation

GNE684 inhibited TNF-driven cell death effectively in several human and mouse cell lines (Fig. 2a and S2a, b). Accordingly, GNE684 disrupted TBZ-induced RIP1 autophosphorylation, interactions between RIP1 and RIP3, RIP3 autophosphorylation, and phosphorylation of MLKL by RIP3 (Fig. 2b, c and S2c). Consistent with observations in cells expressing kinase-dead RIP1 mutant D138N [12, 45], GNE684 did not affect RIP1 protein abundance or TNF-induced activation of NF-κB or MAPK (JNK) signaling, even at elevated concentrations (20 and 200 μM) (Fig. 2d and S2d). GNE684 also blocked TBZ-induced necroptosis in primary human, monkey, and pig colon and stomach, and mouse colon cells, and TB-induced apoptosis in mouse esophagus and small intestine cells (Fig. 2e). Thus, GNE684 is an efficient inhibitor of RIP1 kinase mediated necroptotic and apoptotic cell death in many species with no effect on TNF-induced NF-κB or MAPK signaling.

### Inhibition of RIP1 does not affect the growth of pancreatic tumors

**Fig. 3:**
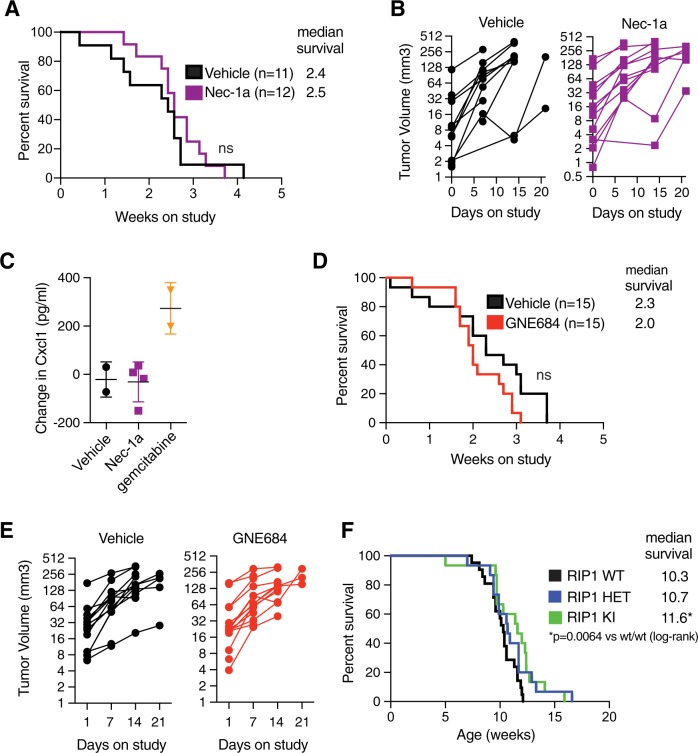
Inhibition of RIP1 does not affect pancreatic tumor growth. **a** Overall survival of *Kras* mutant genetically engineered mouse (GEM) model of pancreatic ductal adenocarcinoma (KPP; *LSL-Kras*^*G12D/+*^*; p16/p19*^*fl/fl*^*; Pdx1-cre*) on continuous Nec-1a treatment (*n* = 11) and vehicle control (*n* = 12); log-rank, NS– not significant, *p* = 0.47. **b** Tumor volume measurements based on serial ultrasound imaging of tumors in mice from **a**. **c** Serum Cxcl1 measurements via Luminex from KPP mice acutely treated (d7) with indicated compounds. **d** Overall survival of KPP model on continuous RIP1 inhibition with GNE684 (*n* = 15) and vehicle control (*n* = 15); log-rank, NS– not significant, *p* = 0.08. **e** Tumor volume measurements based on serial ultrasound imaging of tumors in mice from **d**. **f** Overall survival of KPP animals that were *Ripk1*^*D138N/+*^ (RIP1 HET, *n* = 15), *Ripk1*^*D138N/D138N*^ (RIP1 KI, *n* = 15) or *Ripk1*^*+/+*^ (RIP1 WT, *n* = 21); log-rank, *p = 0.0064

The kinase activity of RIP1 was reported to limit anti-tumor immunity in models of pancreatic ductal adenocarcinoma (PDAC) [25, 26]. We examined the role of RIP1 in two different genetically engineered mouse models of PDAC [41]. Inhibition of RIP1 using Nec-1a in a *Kras* mutant PDAC model (KPP; *LSL-Kras*^*G12D/+*^*; p16/p19*^*fl/fl*^*; Pdx1-cre*) after tumors were established had no impact on overall survival or tumor growth (Fig. 3a, b and S3a). Moreover, in contrast to reported data [25], treatment with Nec-1a had little effect on levels of the chemokine Cxcl1 (Fig. 3c and S3b). GNE684 also had no impact on overall survival or tumor growth in the KPP or KPR (*LSL-Kras*^*G12D/+*^*; p16/p19*^*fl/wt*^*; Trp53*^*R270H/wt*^*; Pdx1-cre*) PDAC models (Fig. 3d, e and S3c–f). Interestingly, genetic inactivation of RIP1 caused a small, but significant increase in overall survival in the KPP model, suggesting a tentative role for the kinase activity of RIP1 in tumor initiation rather than progression (Fig. 3f).

We also explored the claim that inhibition of RIP1 reprograms myeloid cells [26]. In contrast to the reported data, we found no evidence that inactivation of RIP1 in macrophages altered gene expression programs or phosphorylation of STAT1 (Fig. S4). Collectively, these data indicate that inhibition of RIP1 in established tumors has no effect on tumor growth or survival in PDAC models.

### Neither inactivation of RIP1 nor loss of RIP3 impairs metastasis of B16 melanoma cells

**Fig. 4:**
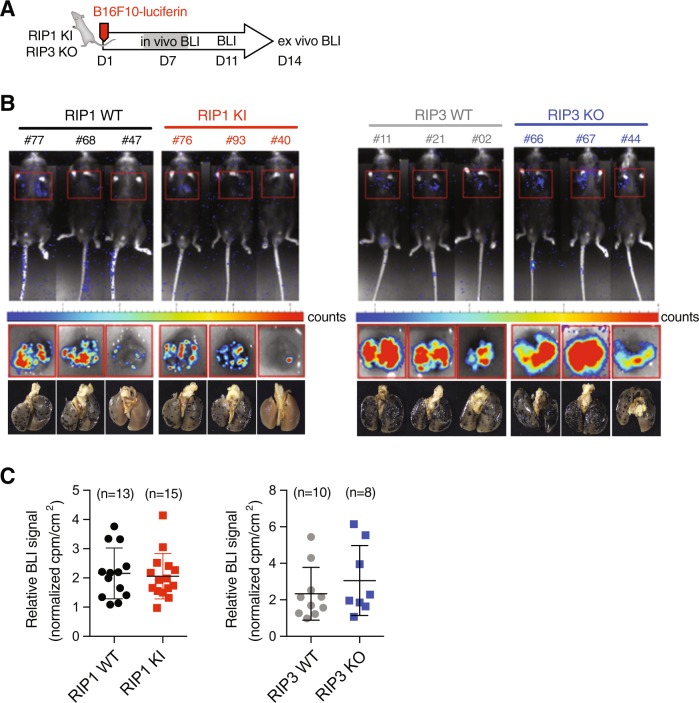
Neither RIP1 inactivation nor RIP3 loss affects B16 lung metastases. **a** Scheme of experimental procedure for luciferin-B16F10 tail vein injection model of metastasis. **b** Representative images of *Ripk1*^*+/+*^ (RIP1 WT), *Ripk1*^*D138N/D138N*^ (RIP1 KI), and *Ripk3*^*+/+*^ (RIP3 WT) or *Ripk3*^*-/-*^ (RIP3 KO) recipients following luciferin exposure on day 14. Either whole animal (upper panels) or ex vivo lung (middle panels) images were collected with corresponding light images (bottom panels). Wild-type littermates were used as controls. **c** Quantitation of ex vivo luciferase imaging of lungs 14 days post injection of B16F10-luciferin cell line in either *Ripk1*^*D138N/D138N*^ (RIP1 KI, red squares, *n* = 15) or *Ripk3*^*-/-*^ (RIP3 KO, blue squares, *n* = 8) recipients

Activation of RIP1 has been implicated in the metastasis of tumor cells [27]. We used B16 melanoma cells expressing luciferase in order to better quantitate cell seeding in the same tail vein injection model (Fig. 4a). Recipient animals lacking RIP3 or expressing kinase-dead RIP1 D138N exhibited comparable cell seeding in the lungs to wild-type siblings (Fig. 4b–d). Therefore, neither RIP3 nor the kinase activity of RIP1 is necessary for melanoma cells to seed the lungs.

### Inactivation of RIP1 prevents colitis and ileitis induced by NEMO deficiency in IECs

**Fig. 5:**
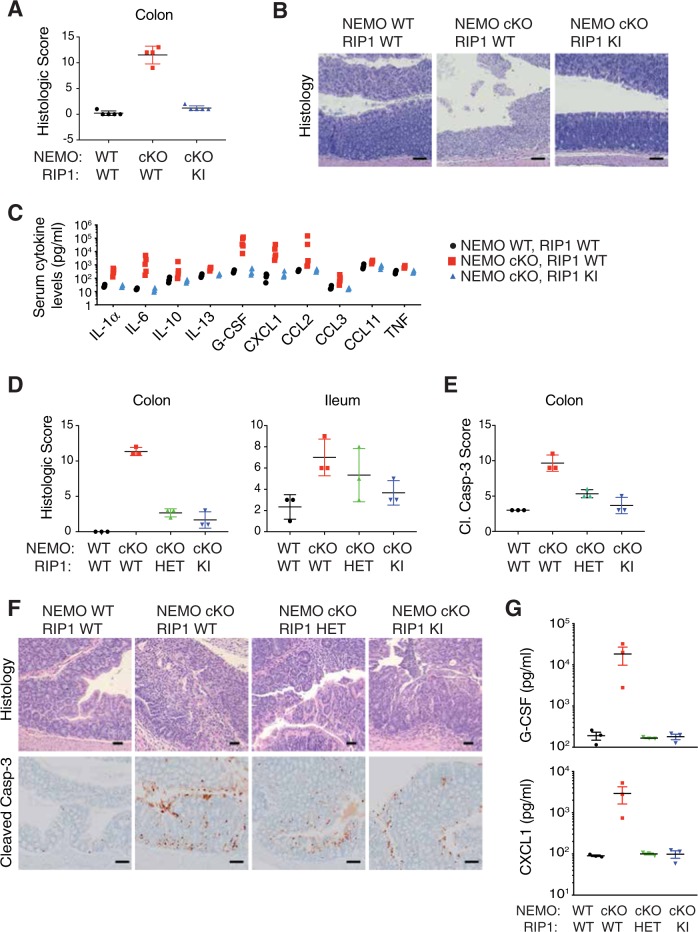
RIP1 inactivation blocks colitis and ileitis induced by NEMO deficiency in IECs. **a** *Nemo*^*+/+*^ *Villin*.creERT2 (NEMO WT, RIP1 WT), *Nemo*^*fl/fl*^ *Villin*.creERT2 (NEMO cKO, RIP1 WT) or *Nemo*^*fl/fl*^ *Villin*.creERT2 *Ripk1*^*D138N/D138N*^ (NEMO cKO, RIP1 KI) mice were treated with tamoxifen on days 1–3 to induce NEMO deletion. Graph depicts histology score of colon sections. **b** Representative images of colon sections stained with H&E from animals analyzed in **a**. Bars = 100 µm. **c** Cytokine and chemokine levels from the serum of mice analyzed in **a** were assessed by Luminex ELISA. **d** Histology scores for mice of indicated genotypes that were either NEMO WT or NEMO cKO and RIP1 WT, *Ripk1*^*D138N/+*^ (RIP1 HET), or *Ripk1*^*D138N/D138N*^ (RIP1 KI). **e** Extent of cleaved caspase-3 in mice of indicated genotypes. **f** Representative images of colon sections of mice analyzed in **d** and **e**. H&E bars = 50 µm, IHC bars = 100 µm. **g** G-CSF and CXCL1 levels from the serum of mice analyzed in **d** was assessed by Luminex ELISA

NEMO deficiency in IECs is reported to cause cell death and inflammation in the ileum and colon in a RIP1 kinase-dependent fashion [17]. We confirmed that acute *Nemo* deletion in IECs (NEMO cKO) caused colitis, and this coincided with increased cleavage of caspase-3 in the colon, a marker of apoptosis, as well as elevated serum cytokines and chemokines (Fig. 5a–d). Inflammation was completely prevented in mice expressing inactive RIP1 D138N (RIP1 KI) (Fig. 5a–c). Interestingly, mice heterozygous for kinase-dead RIP1 (RIP1 HET) showed reduced apoptosis in the colon after deletion of *Nemo*, reduced serum cytokines and chemokines, and almost complete protection from colitis (Fig. 5d–g and S5). Therefore, even partial inhibition of RIP1 can ameliorate cell death and inflammation in this model of inflammatory bowel disease.

**Fig. 6:**
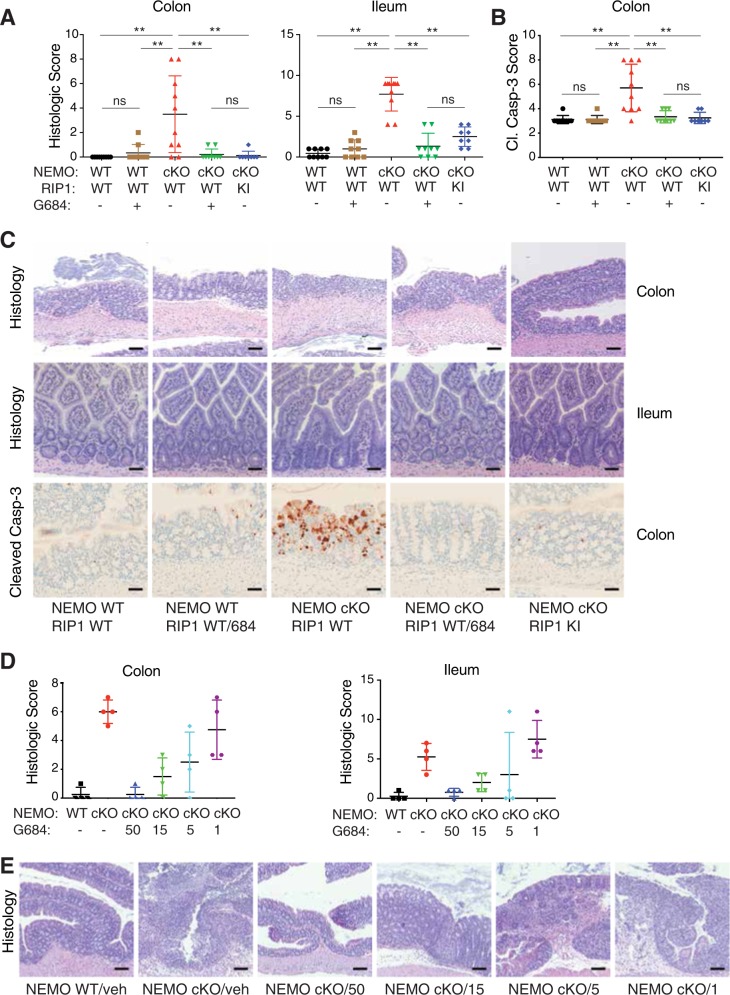
GNE684 inhibits colitis and ileitis caused by NEMO deficiency in IECs. **a** Mice of indicated genotypes were treated with tamoxifen on days 1–3 and with GNE684 (50 mg/kg, PO, BID; 684) from days 2–6. Graphs depict histology score of colon and ileum sections. **b** Extent of cleaved caspase-3 in colon sections of indicated genotypes and treatments. **c** Representative images of colon and ileum sections analyzed in **a** and **b**. Colon bars = 100 µm, H&E ileum bars = 50 µm, IHC bars = 50 µm. Asterisks indicate *p* < 0.01, ns–not significant. **d** Wild-type (WT) or NEMO cKO mice were treated with tamoxifen as in **a** and with indicated doses of GNE684 (mg/kg, PO, BID; 684) from days 2–6. Graphs depict histology score of colon and ileum sections. **e** Representative images of colon sections analyzed in **d**. Bars = 100 µm

Next, we tested whether inhibition of RIP1 with GNE684 prevented disease in NEMO IEC cKO mice. Note that GNE684 alone had no adverse effects on the intestines of wild-type mice (Fig. 6a–c). Dosing with 50 mg/kg GNE684 almost completely protected the NEMO-deficient intestines from colitis and ileitis, and this coincided with reduced apoptosis of IECs and reduced serum cytokines (Fig. 6a–c and S6a, b). Protection was also observed with 15 or 5 mg/kg GNE684, whereas 1 mg/kg GNE684 had a modest effect in the colon but not the ileum (Fig. 6d, e and S6c). Therefore, inhibition of RIP1 with GNE684 affords dose-dependent protection from IEC death and associated inflammation after loss of NEMO.

### Inhibition of RIP1 ameliorates arthritis and skin inflammation

**Fig. 7:**
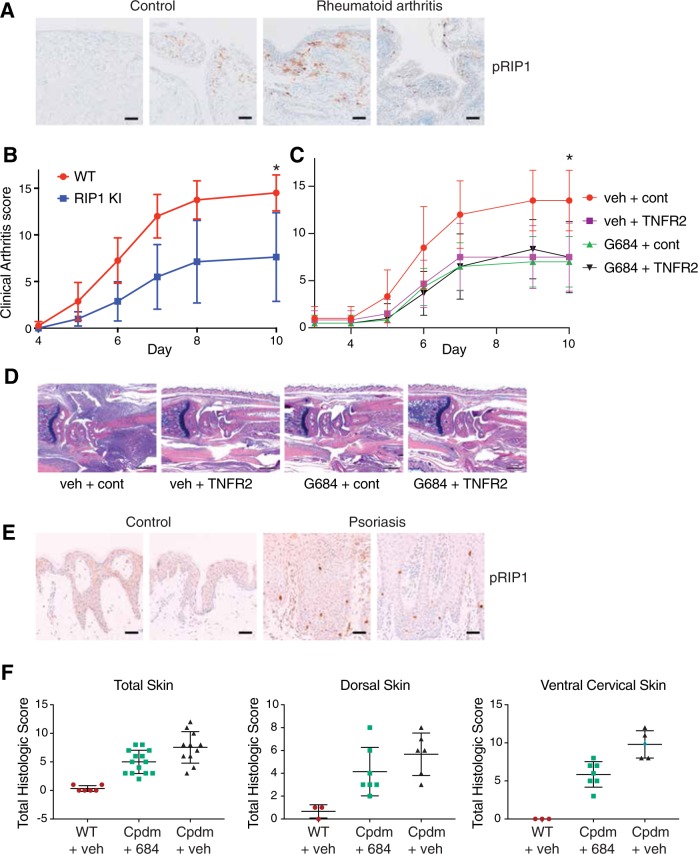
Inactivation of RIP1 reduces arthritis and skin inflammation. **a** Sections of synovium from patients with rheumatoid arthritis (RA) and control human joints were immunolabeled with pRIP1 antibody; representative images are shown. Bars = 100 µm. Out of 20 examined RA samples, moderate to extensive labeling for pRIP1 was observed in 7/20 samples, rare to mild labeling in 8/20 samples and no labeling in 5/20 samples. Labeling for pRIP1 was also observed in 1/7 (moderate) and in 5/7 (rare to mild) normal samples. **b** Wild-type (WT) or *Ripk1*^*D138N/D138N*^ (RIP1 KI) mice (*n* = 6 per group) were injected with 2 mg of a cocktail of anti-collagen antibodies on day 0 and monitored for 10 days. The scoring is a composite of all four paw scores. **c** Wild-type mice were treated as in **b**. On days 4–9 animals (*n* = 7 per group) were administered with vehicle and anti-ragweed-IgG2a (150 μg; cont), vehicle and mTNFR2-IgG2a (150 μg; TNFR2), GNE684 (50 mg/kg, PO, BID; 684) and anti-ragweed-IgG2a (150 μg; cont), or GNE684 and mTNFR2-IgG2a, and scored as in **b**. Asterisks indicate *p* < 0.05. **d** Histology of representative forepaws from **c**. Bars = 500 µm. **e** Sections of psoriatic and normal human skin were stained with pRIP1 antibody; representative images are shown. Bars = 50 µm. Immunolabeling for pRIP1 was observed in 4 out of 20 psoriasis samples, and in none of four normal samples. **f** Wild-type or Sharpin mutant (*Cpdm*) mice treated with vehicle (*n* = 9 for WT and *n* = 7 for *Cpdm*) or GNE684 (50 mg/kg, PO, BID, *n* = 7; 684) for 4.5 days. Dorsal and ventral cervical skin tissues were scored separately and added for the total skin histologic score

To investigate if RIP1 is activated in other inflammatory diseases, we performed immunohistochemistry with an antibody that recognizes human RIP1 autophosphorylated on Ser166 (pRIP1) [29]. HT29 cells treated with TBZ to induce necroptosis labeled strongly for pRIP1, whereas rare to no labeling was observed in untreated cells (Fig. S7a). Interestingly, pRIP1 was also detected in endothelial cells in patient samples exhibiting acute myocardial infarction (Fig. S7b). Both RIP1 and RIP3 have been shown to play a role in myocardial infarction in animal models [22, 46], so these kinases may exert their effects in endothelial cells. Autophosphorylated RIP1 was also detected in human synovium samples from patients with rheumatoid arthritis (RA), with immunolabeling primarily in the subsynovial connective tissue (Fig. 7a).

We next evaluated the role of the kinase activity of RIP1 in a mouse model of arthritis that is induced by anti-collagen antibodies [40]. Antibody injection caused subacute neutrophilic polyarthritis with concomitant cartilage injury and bone remodeling (Fig. 7b). Mice expressing catalytically inactive RIP1 D138N had reduced arthritis in paws and joints compared to wild-type mice (Fig. 7b). GNE684 reduced arthritis in wild-type mice to a similar extent, with protection comparable to that seen using TNFR2-Fc to block signaling by TNF (Fig. 7c, d). A combination of GNE684 and TNFR2-Fc did not reduce arthritis severity further (Fig. 7c, d). Thus, RIP1 may be activated by TNF to promote arthritis in this mouse model.

We also noted that pRIP1 immunolabelling was increased in the epidermis of human psoriasis samples when compared to control skin (Fig. 7e). Consistent with active RIP1 contributing to inflammation in the skin, *Cpdm* mice that are deficient in SHARPIN develop severe skin inflammation unless they also express inactive RIP1 D138N [19]. We found that 6-week-old *Cpdm* mice, which have already developed prominent dermatitis, responded to a brief 4.5-day treatment with GNE684 and showed a reduction in dermatitis severity, particularly in ventral cervical skin where lesions tended to be the most severe (Fig. 7f and S7c, d). Elevated IgM levels in *Cpdm* mice were also reduced by GNE684 (Fig. S7e). Therefore, GNE684 can effectively ameliorate dermatitis in *Cpdm* mice.

## Discussion

The kinase activity of RIP1 has been implicated in many pathologies characterized by inflammation and tissue damage [47]. Thus, inhibition of RIP1 presents an attractive therapeutic opportunity for the treatment of inflammatory diseases [29]. The kinase domain of RIP1 has unique structural features, which allow the development of selective RIP1 inhibitors [30]. However, interspecies differences in RIP1 mean that inhibitors blocking human RIP1 can be over a hundred-fold weaker against murine RIP1 [30]. Although slightly more potent against human RIP1, GNE684 can also inhibit mouse RIP1 effectively. Coupled with its favorable pharmacological profile in vivo, GNE684 represents an ideal compound for investigating the physiological role of the kinase activity of RIP1 in disease settings [48–52]. GNE684 provided much the same level of protection as genetic inactivation of RIP1 in several inflammatory disease models (TNF-driven SIRS, colitis induced by NEMO deficiency in IECs, and collagen antibody-induced arthritis). Therefore, GNE684 can reliably inhibit RIP1 in physiological settings. Importantly, GNE684 was well tolerated and did not affect the abundance of RIP1, or NF-κB and MAPK signaling, either with or without TNF stimulation. Our results demonstrate the benefit of targeting RIP1 in inflammatory diseases.

Whether RIP1 should be targeted in other disease, such as cancer, is less certain. Recent reports have advocated for RIP1 as a target in pancreatic cancer [25, 26], but we observed no benefit from inhibiting RIP1 in mouse PDAC. It is important to note that the published studies used invasive procedures for tumor cell implantation. In addition, RIP1 was inhibited almost from the time of transplantation. Therefore, in contrast to our intervention study, RIP1 was inhibited in more of a prevention setting. Such strategies for tumor implantation are distinct from autochthonous models, in which fully established tumors are treated as a mean to recapitulate clinical intervention treatment. Indeed, two different inhibitors of RIP1 (Nec-1a and GNE684) failed to slow the growth of established tumors, or prolong survival, in two different PDAC models (KPP and KPR). Furthermore, we did not observe macrophage reprogramming and/or STAT1 activation due to RIP1 inactivation, even when using GSK547 to inhibit RIP1 as in the published study [26]. Similarly, we failed to confirm a reported role for RIP3 or the kinase activity of RIP1 in the metastasis of B16 melanoma cells to the lung following tail vein injection. Collectively, our data question the rationale for targeting RIP1 in cancer.

In contrast, we confirm that inhibition of RIP1 is beneficial in inflammatory disease models affecting the joints, skin, and gut. Detection of autophosphorylated RIP1 in psoriasis and rheumatoid arthritis patient samples indicates that RIP1 is also active in human inflammation. Thus, there is a strong rationale for the development of RIP1-targeting anti-inflammatory therapeutics.

## Supplementary information

Patel684-Revised-Supplement

GNE684-DiscoverX report
