# Supplementary material for: RIP1 inhibition blocks inflammatory diseases but not tumor growth or metastases
Source: Cell Death Differ. 2019 May 17;27(1):161–75. doi: 10.1038/s41418-019-0347-0 (PMC7206119; doi:10.1038/s41418-019-0347-0)
Supplement: Supplementary file 1 — Patel684-Revised-Supplement [file 41418_2019_347_MOESM1_ESM.pdf]

**RIP1 inhibition blocks inflammatory diseases but not tumor growth or metastases**

Patel et al.

**Supplementary Information**

**Supplementary Methods**

**Supplementary Tables 1 and 2**

**Supplementary Figure S1-S7 legends**

## Supplementary Methods

### Immunohistochemistry

Immunohistochemistry was performed on routinely formalin fixed, paraffin embedded tissue sections. Cleaved caspase 3 immunohistochemistry was performed with the Cell Signaling rabbit polyclonal anti-cleaved caspase 3 (Asp175) antibody (Cell Signaling Technologies cat #9661) at a concentration of 0.05 $\mu$g/ml on the Ventana Discovery XT platform with CC1 standard antigen retrieval (Ventana) and the OmniMap detection system with DAB chromogen (Ventana). pRIP1 immunohistochemistry was performed with a rabbit monoclonal anti-phospho-RIP1 (S166) antibody clone D1L3S (Cell Signaling Technologies cat #65746) at a concentration of 0.5 $\mu$g/ml on the Benchmark XT platform (Ventana) with CC1 standard antigen retrieval, goat Ig block, and Optiview amplification and detection system with DAB chromogen (Ventana). Human tissue samples were vendor acquired and were confirmed to have histologic changes consistent with their clinical diagnosis. Similar to the histology scores, cleaved caspase 3 scoring of the small

and large intestine was based on segmentation of small and large intestine and summation of individual scores. Scoring criteria were as follows. Small intestine: 1) Rare crypts contain few (~1-3) immunolabeled cells with irregular distribution of crypt labeling and extensive segments lacking labeling, 2) Mild, multifocal labeling in crypts distributed throughout the length of the small intestine, primarily low numbers of immunolabeled cells observed per crypt, 3) Moderate, multifocal labeling of crypts with increased numbers of crypts containing aggregates of immunolabeled cells, and 4) Marked labeling in an estimated >50% of crypts with some crypts containing substantial aggregates of immunolabeled cells. Large intestine: 1) Individual immunolabeled cells distributed throughout the crypts, 2) Less than 5 foci composed of 1-3 crypts with increased cleaved caspase 3 labeling or 2 or fewer foci with labeling associated with complete crypt destruction and effacement, 3) Greater than 2 larger foci with crypt destruction or greater than 5 small aggregates of crypts with increased labeling, 4) Extensive labeling associated with crypt effacement.

**Bioluminescence Imaging (BLI)**

For *in vivo* BLI, mice were anesthetized in an appropriately sized induction chamber where isoflurane was administered using a vaporizer at ~3-5% + O2 until unconscious (loss of righting reflex). Animals were removed from the induction chamber and injected IP with 250 mg/kg luciferin (200 μL injection volume). The mice were then transferred to a light-tight box for imaging and provided maintenance isoflurane anesthesia (~2-3%) via a nose cone. During image acquisition, the animal was maintained on isoflurane anesthesia via nose cone and its body temperature maintained using a warming

pad. Bioluminescence images were acquired using a cooled intensified CCD camera. Image acquisition time was 2 minutes. For *ex vivo* BLI, animals were euthanized and lungs were removed, placed in a weigh boat, and 100 μL of luciferin solution was ectopically applied for one minute prior to imaging.

**ELISA**

The levels of measured chemokines and cytokines from animal sera were assessed by Luminex assay (Biorad or Millipore).

**Whole blood assay**

Fresh human whole blood was diluted with an equal volume of in RPMI-1640 medium (final 50% diluted blood). The blood was then incubated overnight with a 10-point serial dilution of GNE684 and a stimulation cocktail of TNF (200 ng/ml, R&D) and BV6 (2 μM) or LPS (1 μg/ml, Sigma) and zVAD (20 μM, Bachem). Supernatant fluids were assayed for IL-1α and IL-1β levels using MSD assay plates.

**Protein purification**

Purified recombinant human (residues 2–375), rat (residues 2–373), and mouse (residues 2–374) RIP1 kinase domain proteins were expressed in insect cells and purified as described previously (45). All RIP1 proteins included amino-terminal hexa-histidine and glutathione S-transferase tags.

**RIP1 kinase assay**

RIP1 catalytic activity was measured using the Transcreener® ADP2 detection assay kit (BellBrook Labs; Madison, WI) by monitoring substrate-independent RIP1-catalyzed hydrolysis of ATP to generate ADP. All reactions were performed in 50 mM HEPES buffer

(pH 7.5) containing 30 mM $MgCl_2$, 1 mM dithiothreitol, and 0.002% Brij35. Test compounds were added in 10-point titrations with a top concentration of 10 $\mu$M and a final concentration of 0.5% (v/v) DMSO. Forty nL of test compound were incubated with 4 $\mu$L of ATP diluted in the reaction buffer (50 $\mu$M ATP final) in a 384-well black ProxiPlate (Perkin-Elmer). The reactions were initiated by adding 4 $\mu$L of RIP1 diluted in buffer to the plate (0.050 $\mu$M final RIP1). Plates were centrifuged at 1,800 rpm for 30 seconds and incubated for 2 h at room temperature. Controls included blank reactions containing ATP and DMSO, but no RIP1 or test compound, whereas uninhibited control reactions contained ATP, RIP1 and DMSO, but no test compound. Reactions were stopped by addition of stop and detection mix containing 1X Stop buffer, 40 mM EDTA, 27.5 $\mu$g/mL ADP2 antibody, and 4 nM Tracer. Plates were incubated for 1 h at room temperature in the dark. Plates were read on an Infinite® M1000 plate reader (Tecan; Mannedorf, Zurich, Switzerland) with excitation at 635 nm (20 nm bandwidth) and emission at 670 nm (20 nm bandwidth). Fluorescence Polarization (mP) data were converted into ADP concentrations ($\mu$M) using the ADP-ATP standard curve generated by following the protocol in the product manual. Genedata Screener software (Genedata; Basel, Switzerland) was used for the data analysis and to calculate $K_i^{app}$ using the tight-binding quadratic equation (48) and to simultaneously solve for active enzyme concentration $[E]_T$ and $K_i^{app}$ (49). The total active enzyme concentration optimized to between 0.040 - 0.050 $\mu$M.

**SelectScreen and DiscoverX kinase assays**

GNE684 was tested in Thermo Fisher Scientific SelectScreen assay (Madison, WI) against 221 representative kinases at a concentration of 10 μM, which is 476-fold greater than the $K_i^{app}$ for GNE684 against human RIP1 in the RIP1 enzyme assay. The kinase assays were carried out using Z'-LYTE and Adapta activity and LanthaScreen binding assays (Thermo Fisher Scientific, Madison WI) following protocols developed and performed by Thermo Fisher Scientific (https://www.thermofisher.com/us/en/home/products-and-services/services/custom-services/screening-and-profiling-services/selectscreen-profiling-service/selectscreen-kinase-profiling-service.html).

GNE684 was also tested against 468 kinases in the KINOMEscan scanMAX kinase binding assay panel at a concentration of 10 μM following protocols developed and performed by DiscoverX (http://www.discoverx.com/kinase_profiling).

**Cellular necroptosis assays**

GNE684 was tested using established cell lines from human, rat, and mouse to assess its ability to inhibit necroptosis induced by treatment of the cells with a mixture of chemical and biological reagents. Proliferating cells were seeded in a 384-well black, flat, clear-bottom cell culture plate on Day 1 in 25 μL of complete medium (RPMI-1640 for human and rat cells, EMEM for mouse cells) containing 10% heat-inactivated fetal bovine serum (Invitrogen 10082147) and then incubated overnight in a cell culture incubator before adding 3 μL of a mixture of necroptosis-inducing agents to the cells on Day 2. The necroptosis-inducing agents used were: TNFα [tumor necrosis factor-alpha,

recombinant] (human cells received 20 ng/mL human TNFα (R&D Systems 210-TA-005), rat cells received 20 ng/mL rat TNFα (R&D Systems 510-RT-010) and mouse cells received 1 ng/mL mouse TNFα (R&D Systems 410-MT-010)); the broad spectrum caspase inhibitor zVAD [N-benzyloxycarbonyl-Val-Ala-Asp(OMe) fluoromethyl ketone] (Promega PR-G7573) (20 μM); and the inhibitor of apoptosis protein antagonist BV6 [(S,S,2S,2'S)-N,N'-((2S,2'S)-(hexane-1,6-diylbis(azanediyl)bis(3-oxo-1,1-diphenylpropane-3,2-diyl))bis(1-((S)-2-cyclohexyl-2-((S)-2-(methylamino)propanamido)acetyl)pyrrolidine-2-carboxamide)] (2 μM for human and rat cells cells) (Genentech). The cells were then immediately treated in duplicate with a 10-point titration of up to 20 μM GNE684 (or DMSO vehicle) added in a 0.055-μL treatment volume per well using an Echo® acoustic liquid dispenser (Labcyte Inc.; San Jose, CA). The final concentration of DMSO in all wells was 0.2% (vol/vol). The cell culture plate was returned to the cell culture incubator for 18 (human and mouse cells) or 24 (rat cells) hours. After the incubation, 28 μL of CellTiter-Glo® reagent (Promega Corp.; Madison, WI) was added to each well and the plate was placed on an orbital shaker for 1 hour. The luminescence of the wells was read using an EnVision Multilabel Plate Reader (PerkinElmer; Waltham, MA). Treatment of cells with the necroptosis inducing agents leads to a near total (approximately 80%) reduction in the CellTiter-Glo readout as a result of a programmed necroptotic cell death response. RIP1 kinase inhibitors can completely prevent this necroptotic cell death and rescue the cell viability to a level equivalent to that of cells that were not treated with any necroptosis-inducing agents. For the analysis of the cellular necroptosis data, the near total cell death

response induced by necroptosis-inducing agents is set to 0% cell viability, whereas total rescue of cell viability due to prevention of cellular necroptosis is set to 100% cell viability. The inhibitor concentration that provides 50% inhibition of necroptosis ($EC_{50}$) is calculated by fitting the % cell viability data to a four-parameter concentration–response equation (smart fit) using Genedata (Basel, Switzerland) software.

**Synthesis of GNE684**

General Chemistry. All commercially available reagents and solvents were used as received. Reactions using air- or moisture sensitive reagents were performed under an atmosphere of nitrogen using freshly opened EMD DriSolv solvents. Reaction progress was monitored by TLC and/or LCMS. Flash chromatography was performed with Isco CombiFlash Companion systems using prepacked silica gel columns (40–60 µm particle size RediSep or 20–40 µm spherical silica gel RediSep Gold columns, or similar columns from other vendors). Preparative reverse phase HPLC purifications were performed on a Varian Prostar instrument, using a Phenomenex Gemini-NX C-18 (3 cm × 5 cm; 5 µM) stationary phase, with 0.1% aqueous formic acid/ acetonitrile or 0.1% aqueous ammonium hydroxide/acetonitrile gradients as the mobile phase (typically 5–85% acetonitrile over 10 min) with a flow rate of 60 ml/min. NMR spectra were measured on Bruker 300, 400 MHz or 500 MHz spectrometers, and chemical shifts were reported in ppm downfield from TMS using residual nondeuterated solvent as internal standards ($CHCl_3$, 7.26 ppm; DMSO, 2.50 ppm; MeOH, 3.31 ppm). The following abbreviations are used: br = broad signal, s = singlet, d = doublet, dd = doublet of doublets, t = triplet, q = quartet, m = multiplet. The purity of final compounds was verified by HPLC to be >95% in

all cases using either of the following methods: 1) Agilent 1200 instrument with an Agilent SB C-18 (2.1 mm × 30 mm; 1.8 µm particle size) stationary phase, and a gradient of water/acetonitrile (5–95% over 10 min; 0.05% TFA in both phases) at a flow rate of 0.4 mL/min. Quantification of target and impurities was done by UV detection at 254 nm; 2) Shimadzu LC-2010A / 2020A instrument with an Ultimate C-18 (3.0 mm × 50 mm; 3 µm particle size) stationary phase, and a gradient of water/acetonitrile (10–80% over 6 min then 80% over 2 min; 0.05% TFA in both phases) at a flow rate of 1.2 mL/min and oven temperature of 40 °C. Quantification of target and impurities was done by UV detection at 254 nm.

**7-methoxy-1-methyl-4,5-dihydro-3H-pyrido[3,4-b]azepin-2-one.** To a solution of 4-[2-methoxy-5-(methylamino)-4-pyridyl]butanoic acid dihydrochloride (54.0 g, 181.7 mmol, PCT Int. Appl. (2018), WO 2018100070) in *N,N*-dimethylformamide (947 mL) was added *N,N*-diisopropylethylamine (102.7 mL, 621.32 mmol) and 2-(3H-[1,2,3]triazolo[4,5-b]pyridin-3-yl)-1,1,3,3-tetramethylisouronium hexafluorophosphate(V) (82.7 g, 217.5 mmol). The reaction mixture was stirred at 25 °C for 2 hours and then diluted with water (3000 mL). The mixture was extracted with ethyl acetate (3 × 500 mL). The combined organic layers were washed with water (2 × 200 mL), brine (300 mL), dried over sodium sulfate and concentrated under reduced pressure. The residue was purified by column chromatography (silica gel, 100-200 mesh, 0 to 50% ethyl acetate in petroleum ether) to afford 7-methoxy-1-methyl-4,5-dihydro-3H-pyrido[3,4-b]azepin-2-one (22.4 g, 60%) as a yellow solid. $^{1}$H NMR (400 MHz, Methanol-*d4*) δ 8.05 (s, 1H), 6.69 (s, 1H), 3.88 (s, 3H),

3.30 (s, 3H), 2.68 (t, *J* =7.2 Hz, 2H), 2.30 – 2.21 (m, 2H), 2.18 – 2.05 (m, 2H). LC-MS $R_T$ =0.703 min, m/z = 207.1 $[M+H]^+$.

**3-iodo-7-methoxy-1-methyl-4,5-dihydro-3H-pyrido[3,4-b]azepin-2-one.** To a solution of 7-methoxy-1-methyl-4,5-dihydro-3H-pyrido[3,4-b]azepin-2-one (35.6 g, 172.6 mmol) in dichloromethane (1068 mL) was added $N^1,N^1,N^2,N^2$-tetramethylethane-1,2-diamine (129.4 mL, 863.1 mmol) at -15 $^o$C, then iodotrimethylsilane (122.8 mL, 863.0 mmol) was added slowly. The mixture was stirred at -15 $^o$C for 2 h and iodine (175.3 g, 690.5 mmol) was added. After stirring at -15 °C for 1 h the reaction mixture was poured into ice water and quenched by addition of saturated aqueous sodium thiosulfate (ca. 500 mL until the color of the mixture changed from brown to light yellow). The mixture was extracted with dichloromethane (3 × 1000 mL). The combined organic layers were washed with brine (1000 mL), dried over sodium sulfate and concentrated under reduced pressure. The residue was suspended in ethyl acetate (50 mL), the solid product was collected by filtration and dried under reduced pressure to afford 3-iodo-7-methoxy-1-methyl-4,5-dihydro-3H-pyrido[3,4-b]azepin-2-one (48 g, 84% crude yield) as a brown solid. Used in the next step without further purification. $^1$H NMR (400 MHz, $CDCl_3$) δ 8.04 (s, 1H), 6.60 (s, 1H), 4.71 – 4.44 (m, 1H), 3.94 (s, 3H), 3.40 (s, 3H), 3.00 – 2.44 (m, 4H).

**3-azido-7-methoxy-1-methyl-4,5-dihydro-3H-pyrido[3,4-b]azepin-2-one.** To a solution of 3-iodo-7-methoxy-1-methyl-4,5-dihydro-3H-pyrido [3,4-b]azepin-2-one (30.3 g, 91.23 mmol) in *N,N*-dimethylformamide (650 mL) was added sodium azide (17.8 g, 273.8 mmol). The reaction mixture was stirred at 30 $^o$C for 14 h and poured into water (1200 mL). The mixture was extracted with ethyl acetate (3 × 300 mL). The combined

organic layers were washed with water (200 mL), brine (200 mL), dried over anhydrous sodium sulfate and concentrated under reduced pressure (below 25 °C) to afford crude 3-azido-7-methoxy-1-methyl-4,5-dihydro-3H-pyrido[3,4-b]azepin-2-one (23 g, 100% crude yield) as brown solid. Used in the next step without further purification. LC-MS $R_T$ = 0.729 min, m/z = 248.0 $[M+H]^+$.

(**S)-3-amino-7-methoxy-1-methyl-4,5-dihydro-3H-pyrido[3,4-b]azepin-2-one and** (**R)-3-amino-7-methoxy-1-methyl-4,5-dihydro-3H-pyrido[3,4-b]azepin-2-one.** To a solution of 3-azido-7-methoxy-1-methyl-4,5-dihydro-3H-pyrido 3,4-]azepin-2-one (22.5 g, 91 mmol) in water (80 mL) and tetrahydrofuran (400 mL) was added triphenylphosphine (38.2 g, 145.6 mmol). The mixture was stirred at 30 °C for 18 h and concentrated under reduced pressure. The residue was purified by flash column (silica gel, 100 - 200 mesh, 0 to 100% ethyl acetate in petroleum ether) to afford 3-amino-7-methoxy-1-methyl-4,5-dihydro-3H-pyrido[3,4-b]azepin-2-one (20.0 g, 99%) as yellow oil. LC-MS $R_T$ =0.442 min, m/z = 222.2 $[M+H]^+$. Chiral purification by supercritical fluid chromatography (column: ChiralPak AD-3 150 × 4.6 mm I.D., 3 um; mobile phase: A: $CO_2$; B: Methanol (0.05% DEA); gradient: from 5% to 40% of B in 5.5 min and hold 40% for 3 min, then 5% of B for 1.5 min; flow rate: 2.5 mL/min; column temperature: 40 °C) afforded (S)-3-amino-7-methoxy-1-methyl-4,5-dihydro-3H-pyrido[3,4-b]azepin-2-one (peak 1, retention time = 4.219 min, $^1$H NMR (400MHz, $CD_3OD$) δ 8.06 (s, 1H), 6.72 (s, 1H), 3.95 - 3.86 (m, 3H), 3.38 - 3.31 (m, 4H), 2.78 - 2.56 (m, 2H),2.36- 2.25 (m, 1H), 1.95 - 1.76 (m, 1H)) (12.2 g, 49%) as a yellow solid and (R)-3-amino-7-methoxy-1-methyl-4,5-dihydro-3H-pyrido[3,4-b]azepin-2-one (peak 2, retention time = 5.274 min, $^1$H NMR (400MHz, Methanol-*d4*)

δ 8.07(m, 1H), 6.72 (s, 1H), 3.96 - 3.86 (m, 3H), 3.39 - 3.33 (m, 4H), 2.79 - 2.59 (m, 2H), 2.37 - 2.22 (m, 1H), 1.95 - 1.82 (m, 1H)) (12 g, 48%) as a brown solid.

**(S)-N-((S)-7-methoxy-1-methyl-2-oxo-2,3,4,5-tetrahydro-1H-pyrido[3,4-b]azepin-3-yl)-5-phenyl-6,7-dihydro-5H-pyrrolo[1,2-b][1,2,4]triazole-2-carboxamide (GNE684).** A mixture of (S)-3-amino-7-methoxy-1-methyl-4,5-dihydro-3H-pyrido[3,4-b]azepin-2-one (9.0 g, 40.7 mmol), (S)-5-phenyl-6,7-dihydro-5H-pyrrolo[1,2-b][1,2,4]triazole-2-carboxylic acid (9.3 g, 40.7 mmol, PCT Int. Appl. (2017), WO WO2017004500), 1-hydroxybenzotriazole (1.1 g, 8.1 mmol) and 1-(3-dimethylaminopropyl)-3-ethylcarbodiimide hydrochloride (9.4 g, 48.8 mmol) in *N,N*-dimethylformamide (270 mL) was stirred at 25 $^{o}$C for 12 h. The reaction mixture was quenched with water (500 mL) and extracted with ethyl acetate (4 × 200 mL). The combined organic layers were washed with brine (200 mL), dried over sodium sulfate and concentrated under reduced pressure. The residue was purified by flash column (silica gel, 100 - 200 mesh, 0 to 70% ethyl acetate in petroleum ether then 30% ethyl acetate in petroleum ether) to afford (5S)-N-[(3S)-7-methoxy-1-methyl-2-oxo-4,5-dihydro-3H-pyrido[3,4-b]azepin-3-yl]-5-phenyl-6,7-dihydro-5H-pyrrolo[1,2-b][1,2,4]triazole-2-carboxamide (GNE684) as a light brown solid (13.5 g, 76%). $^{1}$H NMR (400 MHz, DMSO-$d_6$) δ 8.27 – 8.19 (m, 2H), 7.45 – 7.30 (m, 3H), 7.27 – 7.18 (m, 2H), 6.87 – 6.81 (m, 1H), 5.56 (dd, *J* = 8.3, 5.9 Hz, 1H), 4.33 (dt, *J* = 11.5, 7.7 Hz, 1H), 3.87 (s, 3H), 3.31 (s, 3H), 3.24 – 2.92 (m, 3H), 2.70 (dt, *J* = 10.2, 5.4 Hz, 2H), 2.62 – 2.50 (m, 1H), 2.37 – 2.21 (m, 1H), 2.15 (tt, *J* = 12.5, 5.0 Hz, 1H). LC-MS $R_T$ = 10.66 min, m/z = 433.2 [M+H]$^+$. HRMS (ESI) *m/z*: [M+H]$^+$ Calcd for $C_{23}H_{25}N_6O_3$ 433.1983;

found: 433.1978; Anal. Calcd for $C_{23}H_{25}N_6O_3$: C, 63.88; H, 5.59; N, 19.43. Found C, 61.22; H, 5.39; N, 18.28.

**RIP1 crystallization**

RIP1 crystallization system was developed based on the previously described structure of RIP1 and the necrostatins (32). Human RIP1 kinase domain residues 1-294 with four cysteine to alanine mutations (C34A, C127A, C233A, C240A) was expressed and purified from SF9 insect cells using IMAC affinity and size exclusion chromatography. Isolated RIP1 kinase domain (10 mg/mL) was mixed with 1 mM compound and co-crystallized in 0.1 M Bis Tris Propane buffer (pH 6.5), 0.2 M sodium iodide, 20% PEG3350, followed by cryoprotection with 35% PEG3350. Data were collected to 2.1 Å under cryo cooled conditions (100K) at beamline 22-ID at the Advanced Photon Source. Data were processed with XDS (50), followed by molecular replacement, model building and refinement using the PHENIX package (51) and COOT (52). Final refinement $R/R_{free}$ statistics converged at 20.6% and 24.6%. Ramachandran statistics calculated indicate that 96.4% of residues are in favored conformations, with no outliers. The coordinates and structure factors have been deposited in the Protein Data Bank (www.rcsb.org) under accession code 6NYH.

**Supplementary Tables**

**Table S1: Data collection and refinement statistics for RIP1/GNE684 structure**

| | RIP1 + GNE684 |
|---|---|
| **Data collection** | APS 22-ID |
| Space group | $P2_12_12_1$ |
| Cell dimensions | |
| $a$, $b$, $c$ (Å) | 46.98, 97.03, 125.24 |
| $\alpha$, $\beta$, $\gamma$ (°) | 90, 90, 90 |
| Resolution (Å) | 48.52-2.10 (2.16-2.10) |
| $R_{sym}$ or $R_{merge}$ | 0.106 (0.872) |
| $I / \sigma I$ | 8.8 (1.6) |
| Completeness (%) | 94.4 (96.6) |
| Redundancy | 5.7 (5.7) |
| | |
| **Refinement** | |
| Resolution (Å) | 48.51-2.10 |
| No. reflections (total/test) | 32202/2154 |
| $R_{work}$ / $R_{free}$ | 20.6/24.6% |
| No. atoms | |
| Protein | 4024 |
| Ligand | 64 |
| Ions | 6 |
| Water | 201 |
| $B$-factors | |
| Protein | 40.4 |
| Ligand | 32.4 |
| Ions | 69.7 |
| Water | 39.5 |
| R.m.s. deviations | |
| Bond lengths (Å) | 0.008 |
| Bond angles (°) | 0.94 |

*Values in parentheses are for highest-resolution shell.

**Table S2. GNE684 kinase selectivity.**

Thermo Fisher Scientific SelectScreen of 221 kinases tested at 10 µM of GNE684.

| Kinase | % inhibition at 10µM | Kinase | % inhibition at 10µM | Kinase | % inhibition at 10µM | Kinase | % inhibition at 10µM |
|---|---|---|---|---|---|---|---|
| ACVR1B | -2 | EGFR(T790M,L858R) | 0.5 | MAPKAPK2 | 4.5 | PRKAA1 | -3 |
| ACVR2B | 5 | ERK2 | 1 | MAPKAPK3 | 3 | PhK_gamma1 | 0 |
| AKT1 | 2.5 | EphA1 | 9.5 | MARK1 | 6.5 | PhK_gamma2 | 1 |
| AKT2 | 4.5 | EphA3 | -3.5 | MARK3 | -2.5 | PrKX | 1.5 |
| ALK2 | 2 | EphA7 | -4 | MEK1 | -7.5 | RAF1(Y340D,Y341D) | -2 |
| ARK5 | -6.5 | EphA8 | 5.5 | MEKK2 | 7 | RIPK2 | 2 |
| ASK1 | 4 | EphB1 | 3 | MELK | 13 | RIPK3 | -3 |
| Abl | 2 | EphB3 | 3.5 | MKK6 | -4.5 | ROCK1 | 1 |
| Aurora_A | 3 | ErbB2 | -5.5 | MKNK1 | 0 | ROCK2 | 1.5 |
| Aurora_B | 6.5 | ErbB4 | 4 | MKNK2 | 4 | RSK1 | 4.5 |
| Axl | 4 | FAK | 7.5 | MLK1 | 7.5 | RSK2 | 4 |
| BMPR1A | -4.5 | FGFR1 | -2 | MLK2 | 0 | RSK3 | 6 |
| BRAF | 6.5 | FGFR3 | -1.5 | MRCK_alpha | -0.5 | Ret | 4 |
| BTK | -3.5 | FGFR4 | -6.5 | MSK1 | -4 | Ron | 4.5 |
| Blk | 6.5 | Fes | 3 | MSSK1 | 2.5 | Ros | 3 |
| Bmx | 4.5 | Fgr | 8 | MST1 | 6 | Rse | 5 |
| BrSK1 | 2.5 | Flt1 | 3.5 | MST2 | 5 | SGK1 | -1 |
| Brk | -1 | Flt3 | 10 | MST3 | 4 | SGK2 | 5.5 |
| CAMKK1 | -7.5 | Flt4 | 14 | MST4 | 3.5 | SGK3 | 1.5 |
| CAMKK2 | 0.5 | Frk | 4.5 | MYLK(smMLCK) | -5 | SIK2 | 8.5 |
| CDK1/cyclinB | -0.5 | GRK2 | 5.5 | MYLK3(caMLCK) | -11 | SLK | 0 |
| CDK2/cyclinA | 6.5 | GRK3 | 2.5 | Mer | -5 | SPHK1 | 4.5 |
| CDK5/p25 | 4.5 | GRK5 | 4.5 | Met | -2 | SRPK1 | -0.5 |
| CDK7/cyclinH | 5 | GRK6 | 6.5 | Mink1 | 18.5 | STK16 | -2 |
| CDK8/cyclinC | 4.5 | GSK3_alpha | 5 | MuSK | 14 | STK33 | 0 |
| CDK9/cyclinT1 | 9 | GSK3_beta | 5 | NEK1 | 7.5 | Src | -1 |
| CHK1 | -7 | HIPK1 | 0 | NEK4 | 4.5 | Srm | 9 |
| CHK2 | 5 | HIPK2 | -1.5 | NEK6 | -2 | Syk | -1.5 |
| CK1_alpha1 | 3 | HIPK4 | 11.5 | NEK9 | 1.5 | TAK1-TAB1 | 0 |
| CK1_delta | 0.5 | Hck | 5 | NLK | -0.5 | TAO2 | 1 |
| CK1_epsilon1 | 5 | Hyl | 1 | PAK1 | 2.5 | TBK1 | 1 |
| CK1_gamma1 | 4 | IGF1R | 5 | PAK3 | 6 | TEC | -1 |
| CK1_gamma2 | 2 | IKK_alpha | -3 | PAK4 | 14 | TGFBR1 | 3 |
| CK2_alpha1 | 3.5 | IKK_beta | 8.5 | PAK6 | 4.5 | TNK2 | 2.5 |
| CLK1 | -0.5 | IKK_epsilon | 3 | PASK | 4.5 | TSSK1 | 6 |
| CLK2 | 2.5 | IRAK1 | -10.5 | PDGFR_alpha | 0 | TTK | -2.5 |
| CLK3 | 1.5 | IRAK4 | 5 | PDK1(direct) | 5 | TXK | 5.5 |
| CLK4 | 2.5 | IRR | 12 | PI3K-A | 16 | TYK2 | 3.5 |
| CSF1R | 2 | ITK | 5 | PI3K-G | 13.5 | Tie2 | -4.5 |
| CSK | -1.5 | InsR | 3.5 | PIM1 | 1 | Tpl2 | 7 |
| CaMKI | -5.5 | JAK1 | 0 | PKA | 1 | TrkA | -3 |
| CaMKII_beta | 2.5 | JAK2 | 2.5 | PKC_alpha | 3 | TrkB | -5 |
| CaMKI_delta | 7.5 | JAK3 | 3.5 | PKC_beta1 | 3.5 | WEE1 | 0.5 |
| CamKII_alpha | 6.5 | JNK1_alpha1 | -5.5 | PKC_delta | 8 | WNK2 | -8.5 |
| CamKIV | -1 | JNK2 | -4 | PKC_epsilon | 11.5 | YSK1 | 3.5 |
| DAPK1 | -11.5 | JNK3 | 3 | PKC_eta | 0 | Yes | 5 |
| DCAMKL2 | 2 | KDR | -0.5 | PKC_theta | -5 | ZAK | -0.5 |
| DDR1 | 4.5 | KHS1 | 12 | PKC_zeta | -2.5 | ZAP-70 | 1 |
| DMPK | -3 | Kit | 3 | PKD1 | 3 | ZIPK | 1 |
| DNA-PK | 4 | LIMK1 | 8.5 | PKGI_alpha | 7 | eEF-2K | 0 |
| DRAK1 | 0.5 | LRRK2 | -19.5 | PLK1 | 4 | mTOR | 2 |
| DYRK1A | 2 | LTK | 7.5 | PLK2 | 3.5 | p38_alpha(direct) | 3.5 |
| DYRK3 | 2 | Lck | 5 | PLK3 | 5 | p38_beta | 4.5 |
| DYRK4 | 2 | Lyn | 7 | PRAK | 2.5 | p38_delta | 3.5 |
| EGFR | 2.5 | MAP4K4 | 11.5 | PRK1 | 2.5 | p38_gamma | 7 |
| | | | | | | p70S6K | 10 |

## Supplementary Figure Legends

**Supplementary Figure 1. Chemical structure, physico-chemical properties and analytical spectra, and pharmacokinetics of GNE684.**

**(A)** Chemical structure and physico-chemical properties of GNE684. **B)** NMR $^{1}$H spectral data of GNE684. **(C)** Kinase selectivity of GNE684 determined in the KINOME*scan*™ screening platform (10 $\mu$M testing) at DiscoverX. Follow-up $K_d$ were determined for TKL family LIMK2 (4.1 $\mu$M (200-fold hRIP1 $K_i^{app}$)) and atypical kinase RIOK2 (>10 $\mu$M). **(D)** Overlay of GNE684 and Nec-1a binding to RIP1 kinase domain. Right panel; Crystal structure overlay of GNE684 (green, accession code 6NYH) and Nec-1a (yellow, accession code 4ITH) in the RIP1 kinase domain. Labelled residues (orange) are part of the DLG-out motif and the hinge ribbon is colored cyan. Left panel; Back-pocket view overlay view of GNE684 and Nec-1a. **(E-H)** Pharmacokinetics of GNE684 in C57BL/6 mice following administration of single dose IV (5 mg/kg) and single dose (QD) (10 mg/kg and 50 mg/kg) or two doses (BID) (50 mg/kg BID, total dose of 100 mg/kg) by oral gavage. Total plasma concentration versus time profiles following 5 mg/kg IV and 10 mg/kg PO administration **(E** and **F),** unbound plasma concentration versus time profile of GNE684 following 10 mg/kg PO administration **(G),** and unbound plasma concentration versus time profile of GNE684 following 50 mg/kg QD and BID PO administration **(H).** Oral doses were formulated as aqueous suspensions in 1% methylcellulose; Intravenous doses were formulated in 5:35:60 DMSO:PEG400:water solution. **(I)** Total plasma GNE684 concentration 4 h after oral dosing as indicated, related to data shown in 1G.

**Supplementary Figure 2. GNE684 inhibits RIP1 kinase driven cell death.**

**(A)** Indicated cell lines were treated for 20 hours with increasing amounts of BV6, TNF (20 ng/ml) and zVAD (20 μM) (left panel) with or without GNE684 (20 μM). Cell viability was assessed by CellTiter-Glo assay. **(B)** L929 cells were treated with BV6 (2 μM), TNF (20 ng/ml) and zVAD (20 μM) with or without GNE684 (20 μM) for indicated periods of time. Cellular lysates were examined by Western blotting with the indicated antibodies. Data are representative of three experiments. **(C)** Cell lines were treated with TNF (20 ng/ml) with or without GNE684 (20 μM, upper panels, and 20 or 200 μM, lower panels) for indicated periods of time. Cellular lysates were examined by Western blotting with the indicated antibodies. Data are representative of three experiments.

**Supplementary Figure 3. RIP1 inhibition does not affect pancreatic tumor growth.**

**(A)** Average weekly fold change in tumor volume as determined by serial ultrasound imaging of KPP tumor treated with vehicle or Nec-1a from Fig 3B. **(B)** Serum Cxcl1 measurements via Luminex from KPP mice treated with Nec-1a for 19 days. **(C)** Average weekly fold change as determined by serial ultrasound imaging of KPP tumor treated with vehicle or GDC684 from Fig 3E. Dunnett's, NS – p=0.29. **(D)** Overall survival of *LSL-Kras*$^{G12D/+}$*; p16/p19*$^{fl/wt}$*; Trp53*$^{R270H/wt}$*; Pdx1-cre (KPR)* model of pancreatic ductal adenocarcinoma on continuous RIP1 inhibition with GNE684 (n=7) and vehicle control (n=7); log-rank, NS – not significant, p=0.55. **(E)** Serial ultrasound imaging of tumors from Fig S3D. Dunnett's, NS – p=0.35. **(F)** Average weekly fold

change in tumor volume as determined by serial ultrasound imaging of KPR tumors treated with vehicle or GNE684 from Fig S3E.

**Supplementary Figure 4. RIP1 kinase inactivation or inhibition does not alter gene expression or STAT1 activation.**

**(A)** Bone marrow derived macrophages (BMDMs) from three different wild-type (WT) and *Ripk1*$^{D138N/D138N}$ (RIP1 KI) mice were left untreated or treated with GNE684 (1 μM), GSK547 (1 μM) or IFNβ (50 units) for 16 hours. Cell lysates were examined by western blotting using indicated antibodies, including two different pSTAT1 antibodies. L stands for longer and S for shorter exposure. **(B)** RNA sequencing comparison of wild-type (WT) and *Ripk1*$^{D138N/D138N}$ (RIP1 KI) BMDMs.

**Supplementary Figure 5. RIP1 inactivation blocks cytokine and chemokine production induced by intestinal NEMO deletion.**

IL-6, CCL2 and CCL3 levels from the serum of mice analyzed in 5D was assessed by Luminex ELISA.

**Supplementary Figure 6. GNE684 blocks colitis and ileitis induced by intestinal NEMO deletion.**

**(A)** G-CSF levels from the serum of mice analyzed in 6A was assessed by Luminex ELISA. NS – not significant. **(B)** Total and unbound GNE684 plasma concentrations in WT or NEMO cKO mice (analyzed in 6A) on days 4, 5 and 6 at 4 hours after the last oral

50 mg/kg BID dose. **(C)** Total and unbound GNE684 plasma concentrations 4 hours after the last day (6) of oral dosing at indicated amounts (1-50 mg/kg BID) in NEMO CKO mice.

**Supplementary Figure 7. RIP1 activation in human patient samples and inhibition in arthritis and dermatitis models.**

**(A)** HT29 cells were left untreated or treated with TNF (20 ng/ml), BV6 (2 μM) and zVAD (20 μM) for 3h. Cells were formalin fixed, paraffin embedded and immunolabeled with pRIP1 S166 antibody. Bars = 50 μm. **(B)** Sections of myocardial infarct and normal human hearts were stained with pRIP1 antibody; representative images are shown. Strong labeling for pRIP1 was observed in the 5 of the 11 acute infarct samples, 10-20% of 8 subacute to chronic infarct/fibrosis samples and in none of 12 normal samples. Bars = 100 μm. **(C)** Histology of skin sections from wild-type and *Cpdm* mice treated with GNE684 or vehicle in 7F. Bars = 100 μm. **(D)** Representative *Cpdm* mice treated with GNE684 or vehicle in 7F. **(E)** IgM levels in wild-type and *Cpdm* mice treated with GNE684 or vehicle as described in 7F.

# Figure S1

**A**

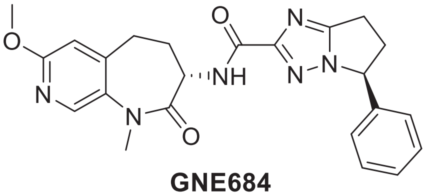

**GNE684**

(S)-N-((S)-7-methoxy-1-methyl-2-oxo-2,3,4,5-tetrahydro-1H-pyrido[3,4-b]azepin-3-yl)-5-phenyl-6,7-dihydro-5H-pyrrolo[1,2-b][1,2,4]triazole-2-carboxamide

Chemical Formula: $C_{23}H_{24}N_6O_3$

Exact Mass: 432.19

ClogP / log$D_{7.4}$ = 2.5 / 1.6

TPSA = 102 Å

Kinetic Solubity ($pH_{7.4}$) = 138.06μM

**B**

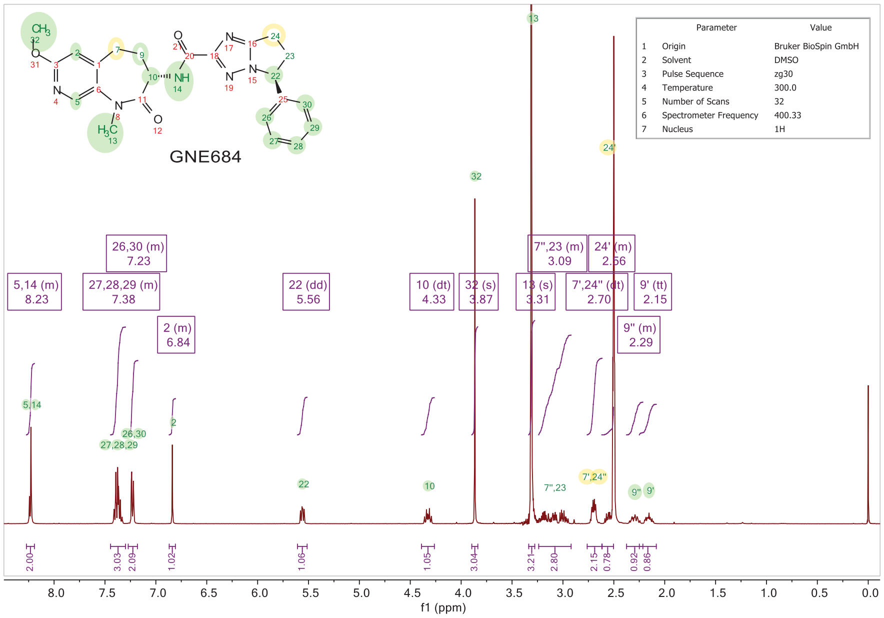


$^1$H NMR (400 MHz, DMSO-$d_6$) δ 8.27 – 8.19 (m, 2H), 7.45 – 7.30 (m, 3H), 7.27 – 7.18 (m, 2H), 6.87 – 6.81 (m, 1H), 5.56 (dd, $J$ = 8.3, 5.9 Hz, 1H), 4.33 (dt, $J$ = 11.5, 7.7 Hz, 1H), 3.87 (s, 3H), 3.31 (s, 3H), 3.24 – 2.92 (m, 3H), 2.70 (dt, $J$ = 10.2, 5.4 Hz, 2H), 2.62 – 2.50 (m, 1H), 2.37 – 2.21 (m, 1H), 2.15 (tt, $J$ = 12.5, 5.0 Hz, 1H).

**C**

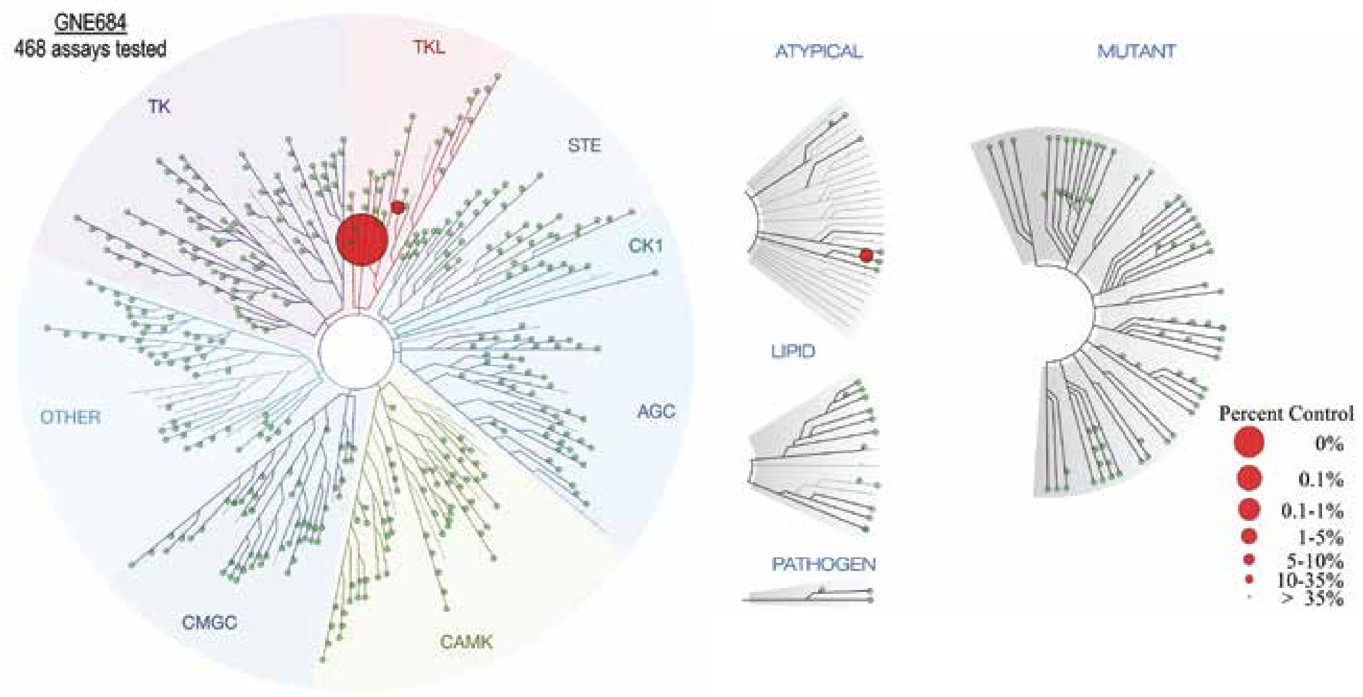


**D**

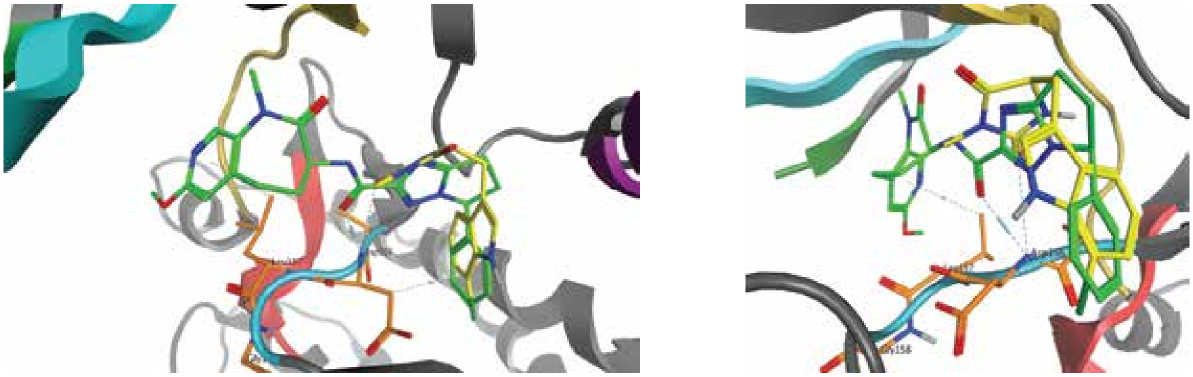

Figure S1

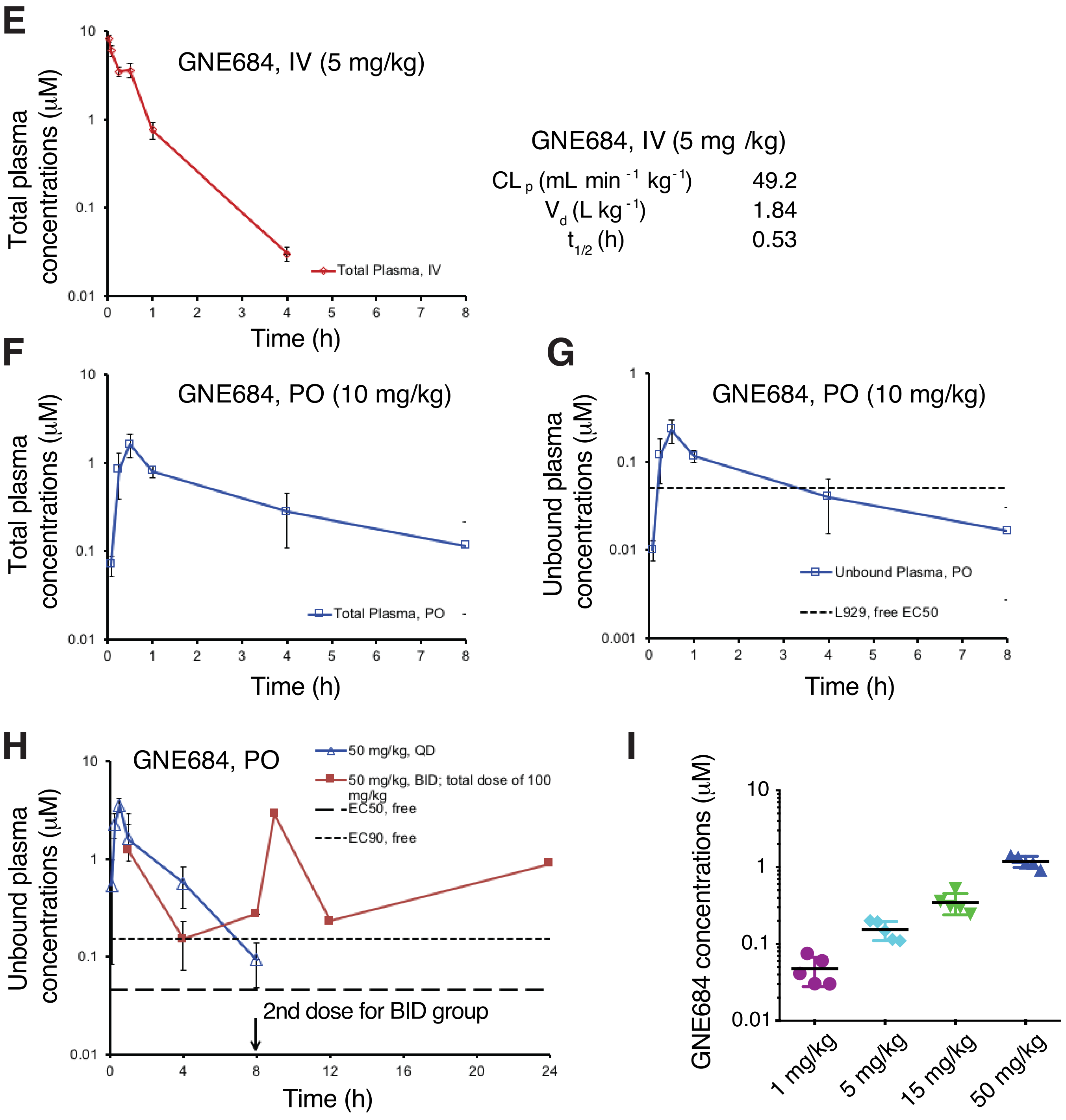

E
GNE684, IV (5 mg/kg)
Total plasma concentrations (μM)
Total Plasma, IV
Time (h)
GNE684, IV (5 mg /kg)
CL p (mL min -1 kg -1) 49.2
Vd (L kg -1) 1.84
t1/2 (h) 0.53
F
GNE684, PO (10 mg/kg)
Total plasma concentrations (μM)
Total Plasma, PO
Time (h)
G
GNE684, PO (10 mg/kg)
Unbound plasma concentrations (μM)
Unbound Plasma, PO
L929, free EC50
Time (h)
H
GNE684, PO
Unbound plasma concentrations (μM)
50 mg/kg, QD
50 mg/kg, BID; total dose of 100 mg/kg
EC50, free
EC90, free
2nd dose for BID group
Time (h)
I
GNE684 concentrations (μM)
1 mg/kg
5 mg/kg
15 mg/kg
50 mg/kg

# Figure S2

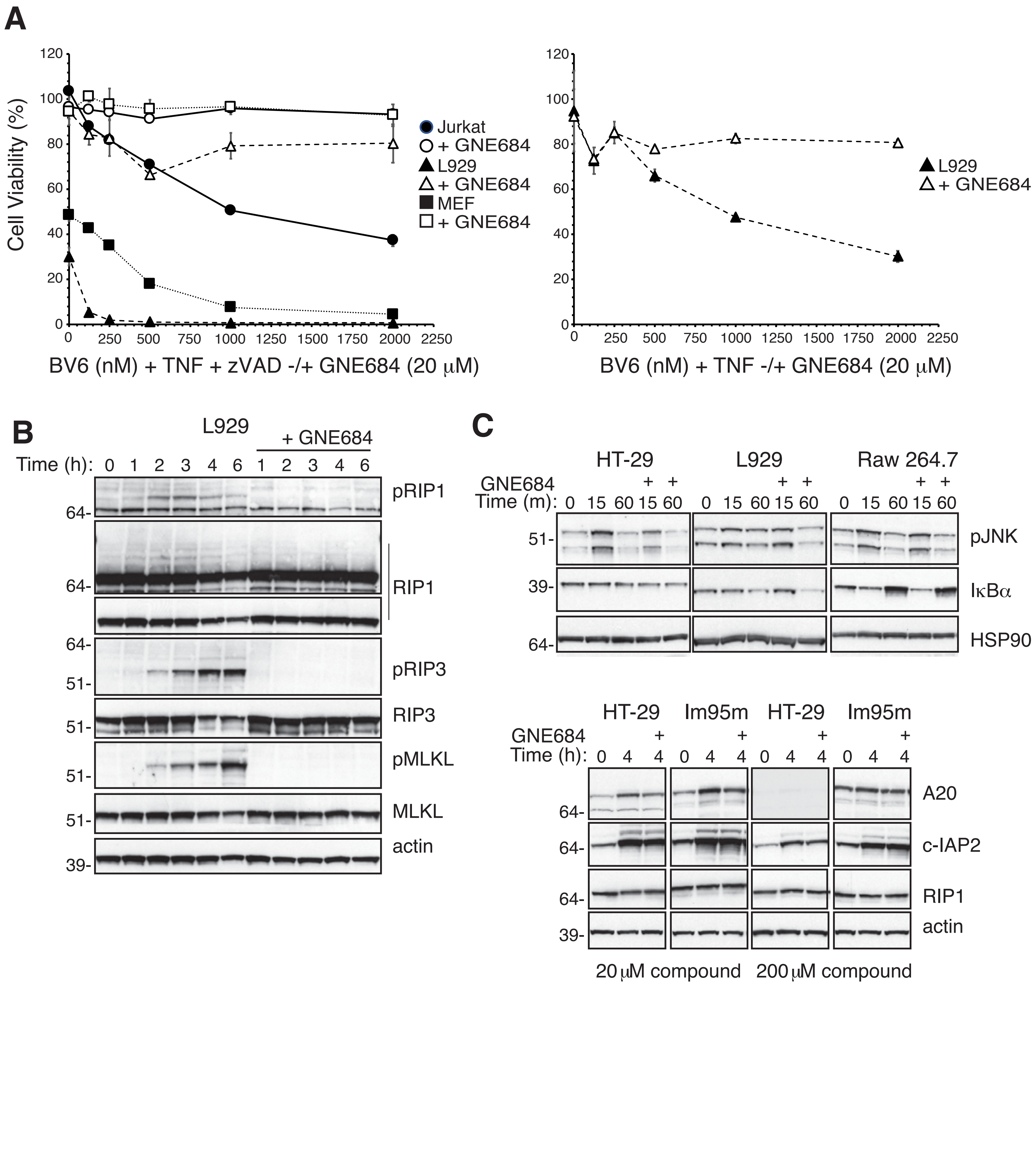
A
Cell Viability (%)
Jurkat
+ GNE684
L929
+ GNE684
MEF
+ GNE684
BV6 (nM) + TNF + zVAD -/+ GNE684 (20 μM)
L929
+ GNE684
BV6 (nM) + TNF -/+ GNE684 (20 μM)
B
L929
+ GNE684
Time (h): 0 1 2 3 4 6 1 2 3 4 6
pRIP1
RIP1
pRIP3
RIP3
pMLKL
MLKL
actin
C
HT-29
L929
Raw 264.7
GNE684
Time (m): 0 15 60 15 60
pJNK
IκBα
HSP90
HT-29
Im95m
HT-29
Im95m
GNE684
Time (h): 0 4 4
A20
c-IAP2
RIP1
actin
20 μM compound
200 μM compound

# Figure S3

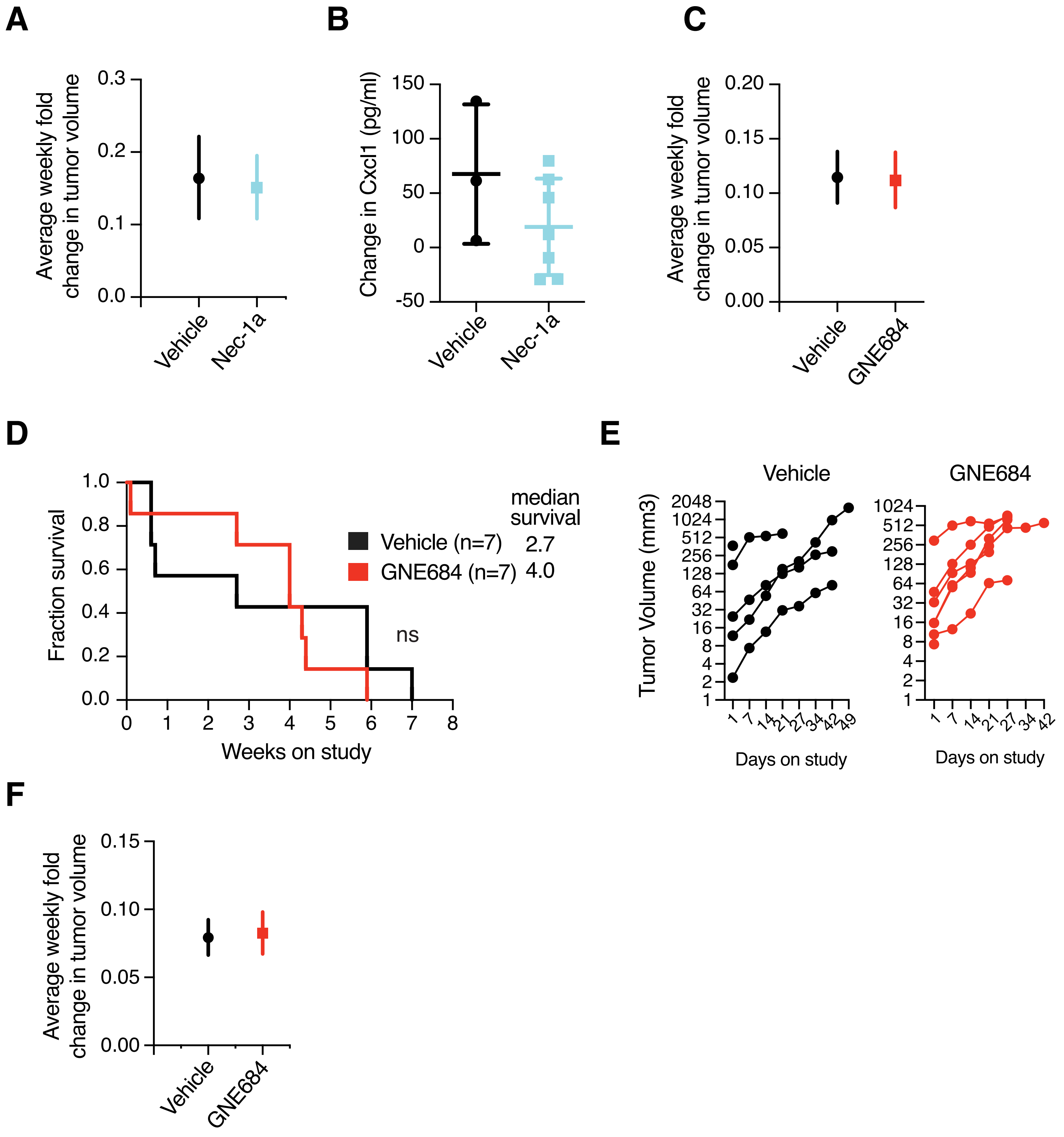
A
Average weekly fold change in tumor volume
0.3
0.2
0.1
0.0
Vehicle
Nec-1a
B
Change in Cxcl1 (pg/ml)
150
100
50
0
-50
Vehicle
Nec-1a
C
Average weekly fold change in tumor volume
0.20
0.15
0.10
0.05
0.00
Vehicle
GNE684
D
Fraction survival
1.0
0.8
0.6
0.4
0.2
0.0
0
1
2
3
4
5
6
7
8
Weeks on study
median survival
Vehicle (n=7) 2.7
GNE684 (n=7) 4.0
ns
E
Vehicle
GNE684
Tumor Volume (mm3)
2048
1024
512
256
128
64
32
16
8
4
2
1
1
7
14
21
27
34
42
49
Days on study
Days on study
F
Average weekly fold change in tumor volume
0.15
0.10
0.05
0.00
Vehicle
GNE684

Figure S4

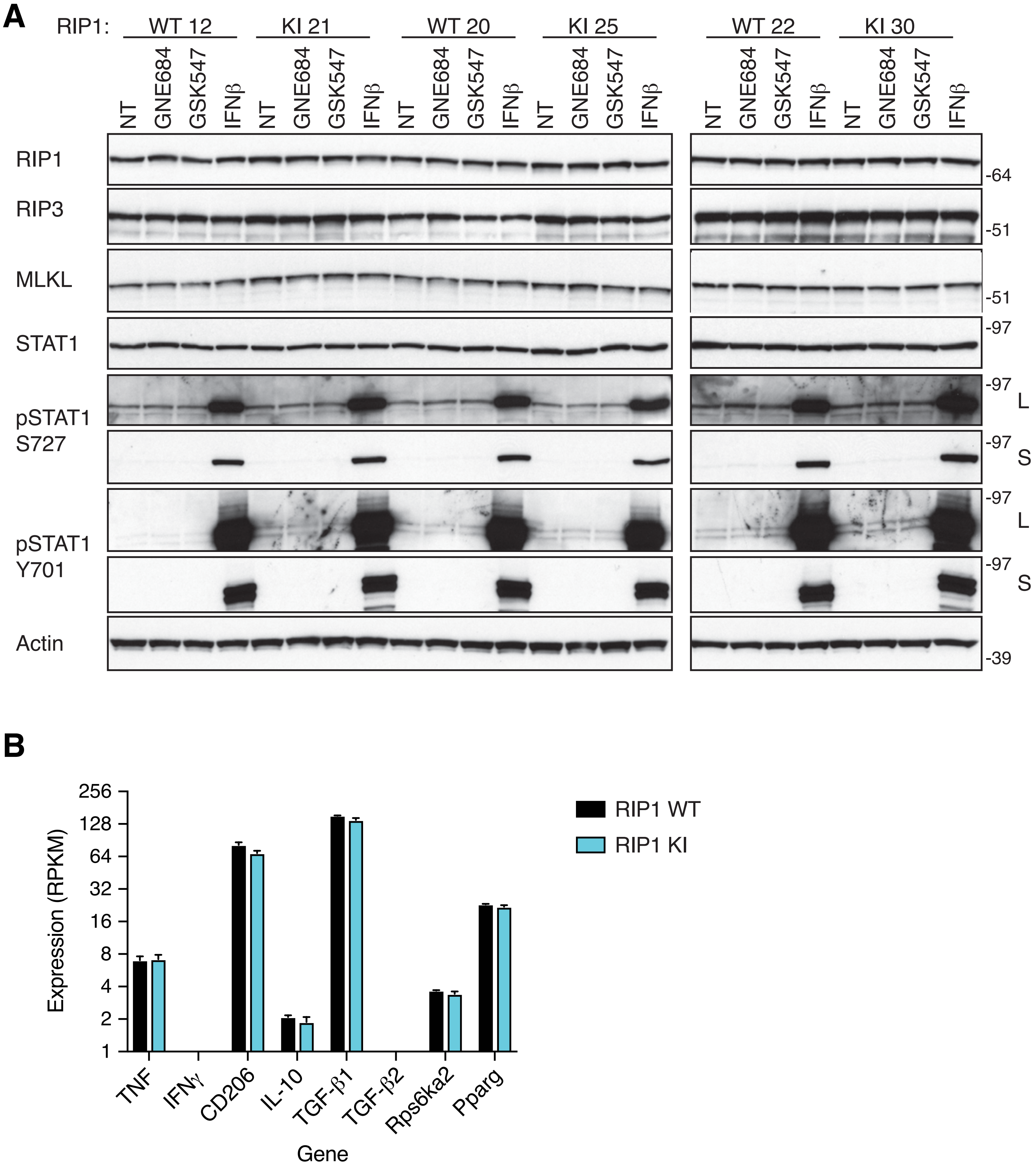
A
RIP1:
WT 12
KI 21
WT 20
KI 25
WT 22
KI 30
NT
GNE684
GSK547
IFNβ
RIP1
RIP3
MLKL
STAT1
pSTAT1 S727
pSTAT1 Y701
Actin
L
S
-64
-51
-97
-39
B
Expression (RPKM)
256
128
64
32
16
8
4
2
1
RIP1 WT
RIP1 KI
TNF
IFNγ
CD206
IL-10
TGF-β1
TGF-β2
Rps6ka2
Pparg
Gene

# Figure S5

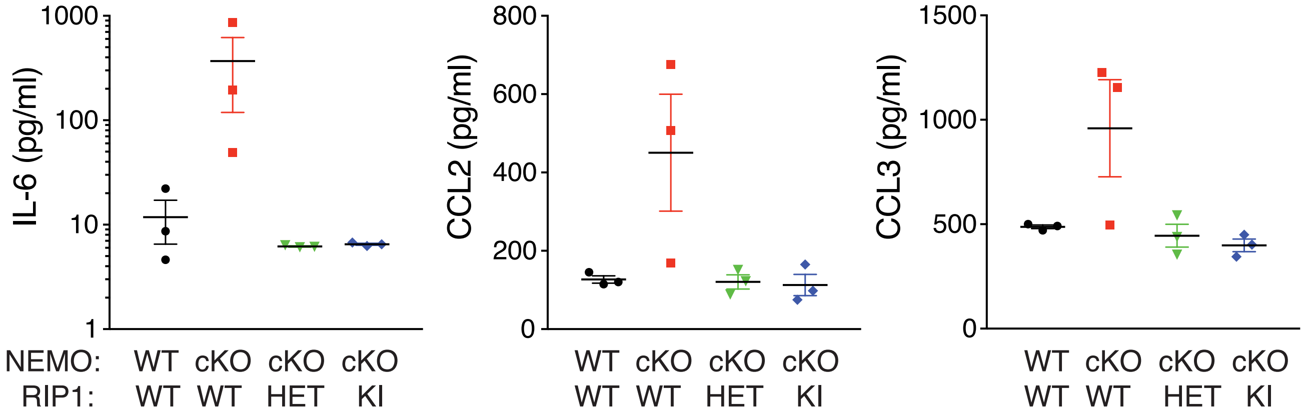

IL-6 (pg/ml)
1000
100
10
1
CCL2 (pg/ml)
800
600
400
200
0
CCL3 (pg/ml)
1500
1000
500
0
NEMO: WT cKO cKO cKO
RIP1: WT WT HET KI
WT cKO cKO cKO
WT WT HET KI
WT cKO cKO cKO
WT WT HET KI

Figure S6

A

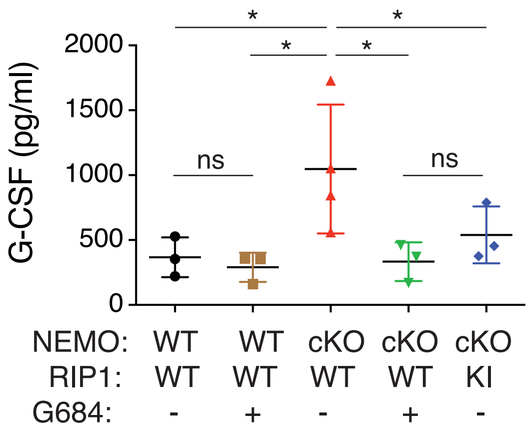


B

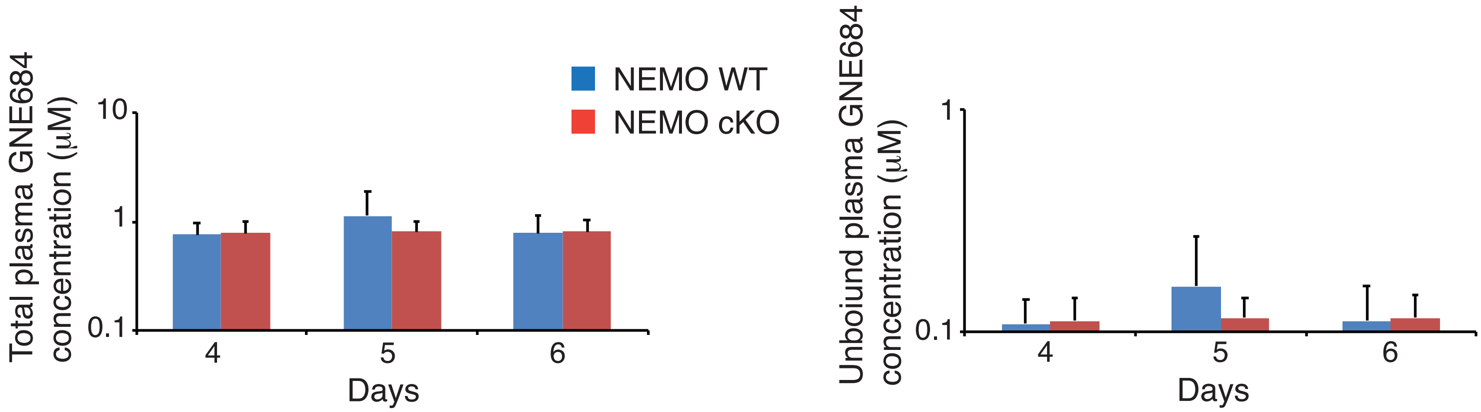


C

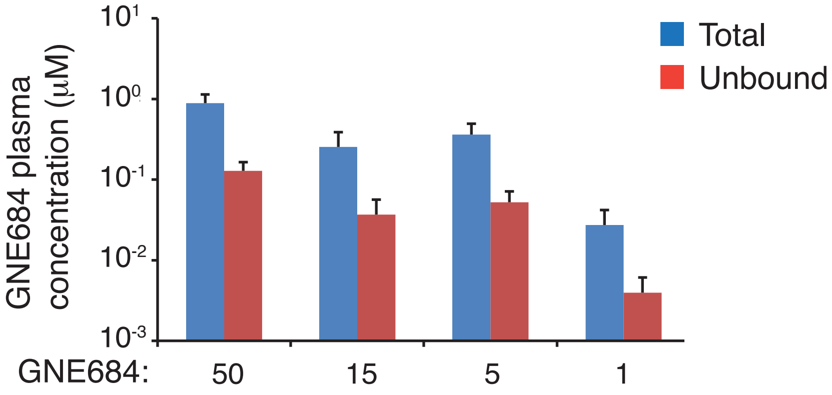

# Figure S7

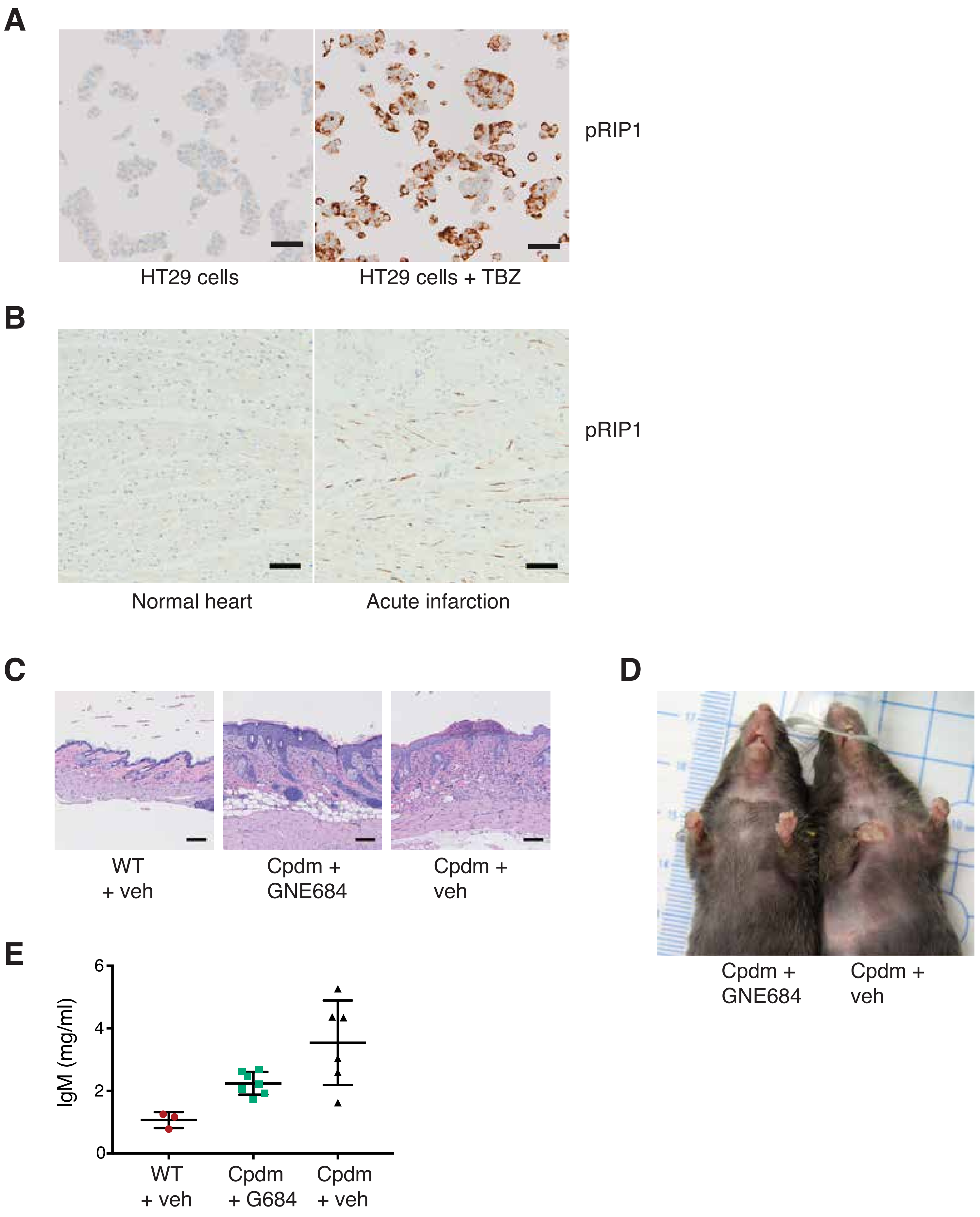
A
pRIP1
HT29 cells
HT29 cells + TBZ
B
pRIP1
Normal heart
Acute infarction
C
WT
+ veh
Cpdm +
GNE684
Cpdm +
veh
D
Cpdm +
GNE684
Cpdm +
veh
E
IgM (mg/ml)
0
2
4
6
WT
+ veh
Cpdm
+ G684
Cpdm
+ veh
